# Pharmacokinetic Drug Interactions of Antimicrobial Drugs: A Systematic Review on Oxazolidinones, Rifamycines, Macrolides, Fluoroquinolones, and Beta-Lactams

**DOI:** 10.3390/pharmaceutics3040865

**Published:** 2011-11-18

**Authors:** Mathieu S. Bolhuis, Prashant N. Panday, Arianna D. Pranger, Jos G. W. Kosterink, Jan-Willem C. Alffenaar

**Affiliations:** Department of Hospital and Clinical Pharmacy, University Medical Center Groningen, University of Groningen, PO Box 30.001, 9700 RB Groningen, The Netherlands; E-Mails: p.n.panday@umcg.nl (P.N.P.); a.pranger@umcg.nl (A.D.P.); j.g.w.kosterink@umcg.nl (J.G.W.K.); j.w.c.alffenaar@umcg.nl (J.-W.C.A.)

**Keywords:** pharmacokinetics, drug interaction, antimicrobial drugs, oxazolidinones, rifamycines, macrolides, fluoroquinolones, beta-lactams, cytochrome P450, induction, inhibition

## Abstract

Like any other drug, antimicrobial drugs are prone to pharmacokinetic drug interactions. These drug interactions are a major concern in clinical practice as they may have an effect on efficacy and toxicity. This article provides an overview of all published pharmacokinetic studies on drug interactions of the commonly prescribed antimicrobial drugs oxazolidinones, rifamycines, macrolides, fluoroquinolones, and beta-lactams, focusing on systematic research. We describe drug-food and drug-drug interaction studies in humans, affecting antimicrobial drugs as well as concomitantly administered drugs. Since knowledge about mechanisms is of paramount importance for adequate management of drug interactions, the most plausible underlying mechanism of the drug interaction is provided when available. This overview can be used in daily practice to support the management of pharmacokinetic drug interactions of antimicrobial drugs.

## 1. Introduction

Antimicrobial drugs manifest a wide variety of drug interactions, which can differ greatly in their extent of severity and clinical relevance. Not only co-medication, but also food and herbal medicine can interact with antimicrobial drugs and vice versa. The nature of these interactions can be of pharmacodynamic (PD) and/or pharmacokinetic (PK) origin.

A PD interaction consists of an alteration of a pharmacological response, through either agonism or antagonism, without affecting the kinetics of the drug. In cases of PD interactions, physicians are advised to re-evaluate the benefit-risk ratio of the co-prescribed drug for each individual patient [1]. PK interactions result in an altered disposition of a drug within a patient and can take place at the level of each of four processes influencing drug exposure, *i.e.*, absorption, distribution, metabolism, and excretion, commonly described by the acronym ADME. Historically, the relevance of drug distribution, particularly of protein binding, has been over-emphasized in the assessment of drug interactions, and nowadays the main cause of drug-drug interactions has been recognized to be modulation of the activity, *i.e.*, inhibition or induction, of cytochrome P450 (CYP) enzymes and transporters.

Clinicians, prescribing the drug and pharmacists—often involved in medication review, therapeutic drug monitoring (TDM), or consultation on drug choice or dose—should be aware of clinically relevant interactions between antimicrobial drugs and co-medication, herbal medicine, and/or food in order to avoid toxicity, side effects, or inadequate treatment. PK interactions are in most cases manageable by adjusting the dose and by monitoring of drug levels (TDM) or vital signs. This review article will address PK interactions of antimicrobial drugs. The scope is to present an overview of PK studies on drug-drug and drug-food interactions of commonly prescribed antimicrobial drugs in daily clinical practice, *i.e.*, oxazolidinones, rifamycines, macrolides, fluoroquinolones, and β-lactam antimicrobial drugs.

## 2. Experimental Section

**Figure 1.**
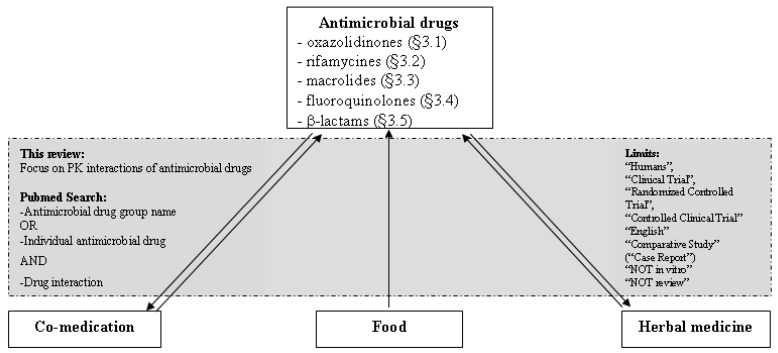
Scope of the review and summary of the experimental section. The gray area symbolizes the focus of this review, *i.e.*, pharmacokinetic (PK) drug interactions of antimicrobial drugs. Arrows symbolize a PK interaction. Upwards pointing arrows (↑): interaction affecting antimicrobial drug (§3.x.1 *“Antimicrobial drugs as victims”*). Downwards pointing arrows (↓): interaction affecting co-prescribed drug (§3.x.2 *“Antimicrobial drugs as perpetrators”*).

The Pubmed database was searched for PK interaction studies on drug-drug and drug-food interactions of antimicrobial drugs (Figure 1). The search was limited through the following selections: “Humans”, “Clinical Trial”, “Randomized Controlled Trial”, “Comparative Study”, and “Controlled Clinical Trial”. Only articles written in English were included. Per group of antimicrobial drugs, a separate search was conducted consisting of the name of the group, the name of the individual drugs, and the term “drug interaction”. When the Medical Subject Heading (MeSH) term “drug interaction” was used, indented terms such as “Herb-Drug Interaction” and “Food-Drug Interaction” were also searched. The search terms “NOT *in vitro*” and “NOT review” were added since this review focuses on original articles of studies with human subjects. Summaries of product characteristics or package leaflets were not consulted since these sources will only present a snapshot of the available information and will therefore not give a good overall impression of their use in clinical practice.

When a search resulted in only a few results, the query was expanded with the criterion “Case Report” and explicitely marked as such in this review since its contents have to be interpreted carefully because of the limited level of evidence.

All searches were conducted in March and April 2011. The relevant results were described per group of antimicrobial drugs. For each group, the drug interactions are divided into interactions affecting the antimicrobial drugs and interactions effecting the co-medication. The drug that is affected is identified by the term ‘victim’ and the drug that causes the effect by ‘perpetrator’. A table summarizing the most important drug interactions is provided for each group of antimicrobial drugs.

## 3. Results and Discussion

### 3.1. Oxazolidinones

Currently, LZD is the only oxazolidinone authorized by the European Medicines Agency (EMA) and the US Food and Drug Administration (FDA). The number of properly designed drug-interaction studies with oxazolidinones is limited and the underlying mechanisms of some drug interactions are not yet fully elucidated. Furthermore, there is a lack of reviewed publications on drug interactions of newer compounds such as PNU-100480, posizolid (AZD2563), radezolid (RX-1741), torezolid and others, several of which are still being studied in phase I, II or III clinical research. A summary of LZD PK interactions is provided in Table 1.

#### 3.1.1. Oxazolidinones as Victims

##### Antimicrobial drugs

In an open-label comparative study of 8 healthy volunteers receiving 600 mg of both LZD and rifampicin (RIF) intravenously, a reduction of LZD plasma concentration was observed [2]. An *in vitro* study demonstrated that LZD is not detectably metabolized by human CYP and did not inhibit the activities of human CYP isoforms 1A2, 2C9, 2C19, 2D6, 2E1, or 3A4 [3]. Based on these observations along with the fact that RIF is a well-known P-gp inducer, the authors suggest LZD to be a P-gp substrate [2]. This hypothesis was further supported by a case report of a patient with MDR-TB. This patient received LZD and clarithromycin (CLR), a potent inhibitor of P-gp and a well-known CYP3A4 inhibitor. It was shown that co-administration of CLR with LZD resulted in a markedly increased LZD AUC [4].

The combination of aztreonam and LZD in an open-label cross-over study that included 13 healthy volunteers resulted in a statistically significant, although probably not clinically relevant increase of LZD AUC of approximately 18% [5]. The authors suggest that the mechanism for this interaction is partly explained by a common elimination pathway, *i.e.*, renal excretion. However, the definite mechanism remains unknown.

##### Food and antacids

In a two-phase single-dose open-label cross-over study of 12 healthy volunteers, a fatty meal caused a small but statistically significant reduction of mean LZD plasma concentration [6]. The C_max_ decreased by 23% and t_max_ increased from 1.5 hours to 2.2 hours, probably due to prolonged gastric residence time. An open-label cross-over study in 28 healthy volunteers tested the hypothesis that a disturbed balanced of reactive oxygen species might lower the *in vivo* clearance of LZD by supplementing dietary antioxidants, *i.e.*, vitamin C and E, but concluded there was no significant effect on LZD C_max_ and AUC [7]. This is in line with current literature indicating that supplemented antioxidant vitamins have subtle effects on *in vivo* reactive oxygen species balance [7]. A randomized open-label cross-over study of 17 healthy volunteers showed that the antacid Maalox^®^ has no effect on the PK of LZD [8].

#### 3.1.2. Oxazolidinones as Perpetrators

##### Serotonin reuptake inhibitors

A single randomized controlled trial (RCT) [9] and several case reports [10–23] describe LZD's potential for drug interactions due to its reversible monoamine oxidase-A inhibitor activity. In case reports, serotonic toxicity was observed after co-administration of LZD with drugs that influence serotonin levels like selective serotonin reuptake inhibitors (SSRIs), tricyclic antidepressants, monoamine oxidase inhibitors, and other serotonergic agents such as citalopram, diphenhydramine, duloxetine, fluoxetine, paroxetine, sertraline, trazodone, and venlafaxine. However, one case report presented a depressed patient receiving co-administered mirtazepine and LZD being treated successfully without toxic signs [24]. The RCT focused on the PK interaction of LZD with the over the counter (OTC) sympathomimetic drugs pseudoephedrine and phenylpropanolamine. A slight increase in blood pressure and a minimal effect on the PK of both co-administered drugs was found in 42 healthy individuals [9]. The serotonin reuptake inhibitor dextromethorphan was co-administered with LZD with no clinical effect: only a slight decrease of dextrorphan, the primary metabolite of dextromorphan, was observed [9].

### 3.2. Rifamycines

The antimicrobial group of rifamycines exhibits their bactericide effect through inhibition of bacterial DNA-dependant RNA polymerases. The most pronounced members of the group, rifampi(ci)n (RIF), rifabutin (RFB) and rifapentine (RFP), are often administered for the treatment of *Mycobacterium tuberculosis*, leprosy (Hansen's disease), and *M. avium complex*. A summary of the interactions is presented in Table 2.

#### 3.2.1. Rifamycines as Victims

##### Antiretroviral drugs

Amprenavir co-administered with RFB or RIF in a randomized prospective cohort study that included 24 healthy subjects resulted in a 293% increase of RFB AUC, but had no effect on RIF kinetics [25]. The authors suggest amprenavir both to be a CYP3A4 inhibitor and substrate and RFB as a CYP3A4 substrate being a less potent inducer of CYP3A4 than RIF. Indinavir, a potent inhibitor of CYP3A4, increased the RIF mean AUC by 73% in HIV-infected patients [26]. When the RFB dose was decreased from 300 mg/day to 150 mg/day and the indinavir dose was increased from 2400 mg/day to 3000 mg/day, the combination produced similar indinavir PK parameters but an increase of RFB AUC by 70% [27]. Another study showed that when 300 mg RFB was added to indinavir 2400 mg/day, AUC exposure to RFB significantly increased by 273% [28]. Nelfinavir increased the AUC of RFB by approximately 22% in a prospective cohort of seven tuberculosis patients [29]. RFB AUC increased by 44% and C_max_ by 45% after co-administration with saquinavir and was well tolerated in a randomized open-label study of HIV-infected patients [30]. Co-administration of tenofovir disoproxil fumarate and RIF resulted in no significant changes in PK parameters [31]. Atazanavir co-administration with RIF resulted in an increased RIF exposure by 160–250% [32]. Co-administration of didanosine with RFB resulted in unchanged RFB exposure [33].

##### Antifungal drugs

Fluconazole had little [34] to no [35] effect on RIF PK parameters. However, fluconazole did increase RFB AUC by 82% and its metabolite LM565 by 216%, possibly through inhibition of CYP3A [36]. Posaconazole caused the AUC of RFB to increase by 72% and the C_max_ by 31%, also possibly through CYP3A4 inhibition [37].

##### Antimicrobial drugs

CLR was found to increase RIF AUC by 60% [38] and RFB AUC by 76–99% in two studies [39,40]. Co-trimoxazole increased the median AUC of RIF by 60% and C_max_ by 31% [41]. The bioavailability of RIF was reduced by approximately 32% when co-administered with isoniazide in a fixed dose combination capsule formulation of 450 mg RIF and 300 mg isoniazide, possibly caused by enhanced decomposition of RIF to the insoluble metabolite 3-formyl RIF SV in the gastrointestinal tract due to the presence of isoniazide in the acidic stomach [42]. The RFP exposure was reduced by co-administration with moxifloxacin (MXF), resulting in a decrease of RFP AUC by 20.3% [43]. The authors explain this decrease by autoinduction of metabolism of RFP through high doses of RFP administered intermittently for more than two weeks. One study focused on the effect of pefloxacin on RIF PK and found that the AUC of RIF in serum was increased by 200% [44]. In a randomized single-dose cross-over study of 16 tuberculosis patients, co-administration of RIF and isoniazide with or without pyrazinamide resulted in a significantly reduced AUC and C_max_ of RIF [45].

##### Food and antacids

A randomized cross-over study in 14 healthy subjects found that the antacid aluminum-magnesium did not alter PK parameters of RIF, but a high-fat meal did reduce the C_max_ by 36% and the AUC by a mere 6%, nearly doubling the t_max_ [46]. The effect of food intake on rifalazil PK parameters was studied in 12 healthy volunteers receiving a normal breakfast or a high-fat breakfast, with a fat content of respectively 30% or 60% of the total calories, resulting in a progressively increased exposure by 30% to 60% [47].

#### 3.2.2. Rifamycines as Perpetrators

##### Antidiabetic drugs

The effect of RIF on antidiabetics such as gliclazide, glimepiride, and glyburide has been investigated in several studies. In a randomized cross-over study in nine healthy volunteers, RIF decreased the gliclazide AUC by 70% with clinically significant changed blood glucose levels [48]. The authors hypothesized that the decreased exposure was caused by induction of CYP2C9. A study with a similar design in 10 healthy subjects found the glimepiride exposure to decrease by 34% [49]. In a randomized open-label cross-over study, co-administering glyburide and a single intravenous dose of RIF demonstrated that RIF inhibited organic anion-transporter polypeptide (OATP) family members, which resulted in the glyburide AUC to decrease by 125% and C_max_ by 81% [50]. In the same study multiple oral doses of RIF resulted in a decreased glyburide exposure, probably through induction of liver enzymes. In three cross-over studies of healthy volunteers RIF reduced repaglinide AUC by 31–81%, an effect probably caused by induction of CYP3A4 and possibly by CYP2C8. This resulted in changed blood glucose profiles [51–53]. RIF had no effect on PK parameters of diabecon (D-400), a complex herbal supplement supposedly offering glycemic control [54].

##### Antihistamines

RIF reduced the fexofenadine bioavailability through induction of intestinal P-gp, resulting in an increased oral clearance, calculated from dose/AUC, and a decrease of AUC [55]. RIF altered the disposition and antihistamine effect of ebastine, a non-sedative antihistamine, reducing the AUC of the active metabolite carebastine to 15% and reducing oral bioavailability [56]. The authors suggest that this might be caused by CYP3A4 induction, possibly combined with P-gp or CYP2J2 induction.

##### HMG-coA reductase inhibitors (statins)

RIF reduced exposure to atorvastatin by 80%, probably by CYP3A4 induction [57]. A recent study in 12 healthy Asian volunteers found that a single dose of RIF increased pravastatin AUC_0-∞_ by 182%, possibly through inhibited hepatic uptake by OATPs and inhibited biliary excretion mediated by MRP2 [58]. However, a multiple-dose study in 10 healthy Caucasian volunteers found that the pravastatin AUC was reduced by 31% [59]. This difference might be explained by up-regulation of mRNA of drug transporters, e.g., OATP and CYP3A4, after multiple doses of RIF or possibly by different polymorphism of Asians compared to Caucasian volunteers. RIF had no effect on the mean AUC of rosuvastatin (95.8%), although in some patients a marked decrease was noted [60]. The authors suggest that OATP1B1 transporter expression might vary, resulting in variable intracellular RIF concentration and interpatient variability in AUC. RIF greatly decreased the AUC of simvastatin by 87%, possibly by CYP3A4 mediated first pass effect in the liver and intestinal wall [61]. In a randomized open-label 2-way cross-over study of 10 healthy subjects, pretreatment with 600 mg RIF reduced the 8 mg single-dose rosiglitazone AUC by 65% and C_max_ by 33%, probably through induction of CYP2C8 and, to a lesser extent CYP2C9 [62]. RIF reduced the pioglitazone AUC significantly by 54%, without a significant effect on C_max_, probably via induction of CYP2C8 enzyme system [63]. RIF had a similar effect on nateglinide, reducing the AUC by 24% without affecting the C_max_ [64].

##### Immunosuppressants

Several interaction studies co-administering rifamycins with immunosuppressants have been conducted. In a small study of four patients, RIF significantly decreased cyclosporine A serum trough concentrations through induction of CYP450 enzyme mediated metabolism [65]. A cross-over study in 12 healthy volunteers receiving RIF and everolimus found a reduced everolimus C_max_ by 58%, AUC by 63%, and a reduced t_1/2_ from 32 to 24 hours [66]. Co-administration of RIF with mycophenolic acid (MPA) was investigated in a prospective cohort PK interaction study following 8 stable renal allograft recipients [67]. A decreased MPA AUC by 17.5% was observed, with an increased exposure to the glucuronidated metabolites. This suggested an induction of uridine diphosphate glucuronosyltransferase (UGT), resulting in increased MPA glucuronidation which possibly also was due to RIF-associated increased expression of MRP2. The authors hypothesized that RIF competitively inhibits MRP2-mediated transport of MPA, inhibiting the enterohepatic recirculation. RIF appeared to induce both intestinal and hepatic metabolism of tacrolimus by means of induction of CYP3A and P-gp in the liver and small bowel, resulting in a decreased bioavailability by 35%, along with an increased clearance [68]. RIF had no effect on PK of temsirolimus in healthy volunteers, but resulted in a decrease of C_max_ by 36%, and did reduce the sirolimus AUC by 65% and C_max_ by 56% [69].

##### Anti-retroviral drugs

Amprenavir co-administered with RFB in a randomized prospective cohort study that included 24 healthy subjects resulted in no change in amprenavir PK, whereas in the same study the combination of RIF and amprenavir resulted in a decrease of amprenavir AUC by 82% [25]. Atazanavir combined with RIF was examined in two studies. One study found a minor increase of atazanavir AUC of 10% but a decrease of the C_min_ by 59%. This is clinically relevant as C_min_ is the relevant PK parameter predicting anti-retroviral activity [32]. The second study, combining atazanavir/ritonavir with RIF, was terminated after patients encountered adverse events (*i.e.*, vomiting and elevated transaminaseelevations levels) [70]. RFB increased darunavir/ritonavir exposure by approximately 60% [71]. Delavirdine was studied with both RFB and RIF in 12 HIV-positive patients. RIF co-administration resulted in a 2700% increase of oral clearance, leading to nearly negligible delavirdine concentrations [72]. RFB increased the oral clearance by 500% [73]. Co-administration of didanosine with RFB resulted in a 20% AUC increase of didanosine [74]. Combining RIF with enfuvirtide had no effect on enfuvirtide PK [75]. Efavirenz and RIF co-administration was studied in a non-blinded randomized PK study, resulting in a decrease in mean efavirenz C_max_ by 24% and AUC by 25% [76]. Other studies found either an increase in AUC of 5% [77] or a non-significant difference in the C_min_ [78]. When 700/100 mg fosamprenavir/ritonavir bid was combined with RFB, a dose reduction of RFB by 75% is advised based on a PK interaction study in healthy volunteers [79].

Indinavir is indicated as a substrate of CYP3A4, and ritonavir a potent CYP inhibitor. Co-administration of RIF with indinavir-ritonavir resulted in an AUC decrease of indinavir by 87% and ritonavir by 94%, besides a decrease in rifamipicin exposure [80]. A study combining 600 mg RIF once a day and adjusted dose lopinavir/ritonavir tablets of 600/150 mg or 800/200 mg was terminated due to adverse events, e.g., elevated AST/ALT, nausea and vomiting [81]. When maraviroc 100 mg was combined with RIF, the AUC of maraviroc was decreased by 50% and the C_max_ by 70%, probably through induction of CYP3A4 [82]. Doubling the maraviroc dose to 200 mg resulted in maraviroc exposure approaching pre-induction values. Co-administration of nevirapine with RIF resulted in a decreased AUC by 36% and C_min_ by 32% in one study [83], and a reduced C_min_ by 39% in another [84]. The possible explanation lies in the fact that nevirapine is a CYP3A4 and 2B6 substrate or possibly even a P-gp substrate. When raltegravir 400 mg was co-administered with RIF, the C_min_ was decreased by 61% and the AUC by 40%. After doubling the raltegravir dose the effect of RIF on raltegravir dose was abolished, but the C_12h_ still dropped by 53% compared to raltegravir alone, possibly by potent induction of UGT 1 family, polypeptide 1 (UGT1A1), and CYP3A4 [85]. RIF reduced the AUC of saquinavir when co-administered with soft-capsule saquinavir by 70% in healthy volunteers and by 46% in patients [86]. RFB had a similar effect, decreasing AUC by 47% in HIV-infected patients [30]. Co-administration of tipranavir/ritonavir with a single dose of RFB resulted in a 16% increase in tipranavir C_12h_ in healthy subjects [87]. Tizanidine and RIF co-administration in a blinded randomized cross-over trial of 10 healthy volunteers resulted in a decrease of tizanidine AUC by 54% and C_max_ by 51%, probably mostly through CYP3A4 and to a lesser extent through CYP1A2 induction [88]. RFB was found to have no effect on safety and PK parameters when combined with zidovudine, and thus no effect on UGT1A1 [89,90]. RIF increased apparent oral clearance of zidovudine [91,92], resulting in a decrease of AUC by 47% [91]. The authors suggest the underlying mechanism is an enzyme-inducing effect of RIF on the glucuronidation of zidovudine [91–93].

##### Benzodiazepines

Rifaximin had no effect on PK parameters of midazolam, which is primarily metabolized by CYP3A4 [94]. RIF did have a significant effect on midazolam, decreasing the AUC by approximately 96% and clinically significantly reducing the PD effects [95,96]. Also triazolam, predominantly metabolized by CYP3A4, was affected by RIF, with a reduced AUC by 95% and C_max_ by 88% in a randomized double-blinded cross-over study resulting in an ineffective triazolam treatment [97]. Two different studies found that RIF reduced the AUC of zolpidem by 73% and C_max_ by 58% [98] and of zopiclone by respectively 82% and 71% [99], probably through CYP3A4 induction.

##### Antimycotics

RIF was observed to both inhibit and induce caspofungin disposition, resulting in a reduced C_24h_ by 14 to 31% after multiple RIF doses, and was found to exhibit a transient net inhibition before full induction of metabolism, leading to an elevated AUC of caspofungin by 61% on the first days of co-administration [100]. RIF reduced the fluconazole AUC by 22% [101]. An open-label single-dose study found RIF reduced the itraconazole AUC by 88% in healthy subjects and by 64% in HIV-infected patients [102]. Finally, the posaconazole AUC decreased by 49% and the C_max_ by 43% in 24 healthy volunteers receiving RFB co-medication, possibly through induction of the UGT enzyme system [37].

##### Antimicrobial drugs

No significant PK interaction was found between azithromycin (AZM) and RFB [103]. RFB was observed to decrease CLR AUC by 44%, but increased the AUC of its metabolite 14-OH-CLR in a study including 34 patients [40]. RIF reduced the exposure to co-trimoxazole, *i.e.*, a reduction of trimethoprim of 47% and of sulfamethoxazole of 23% [104]. A study combining doxycyclin with RIF found significantly lower doxycyclin levels, higher clearance, and a significantly reduced t_1/2_ and AUC [105]. Co-administering RFP with MXF resulted in a decrease in AUC of MXF by 17%, besides reducing RFP exposure [43]. When RIF was combined with MXF, the MXF AUC was reduced by 27–31% [106,107]. RIF caused a reduction of pefloxacin AUC by 27% when administered concomitantly [108]. In a three-way randomized cross-over design study, co-administration of RIF with quinine resulted in an increase of unbound clearance, Cl_un_, by 690% [109]. In a different study, RIF reduced the median AUC by 75% through induced metabolic clearance [110]. RIF has no effect on the AUC of dapsone, but increased dapsone clearance by 67% [111].

##### Hormones

Both rifalazil [112] and rifaximin [113] exhibited no effect on PK parameters of ethinyl estradiol after co-administration in healthy volunteers. A blinded randomized placebo-controlled, cross-over study of 19 healthy volunteers found the AUC of toremifene and of tamoxifen to be decreased by approximately 87% and the C_max_ by 55% after co-administration with RIF [114].

##### Anti-hypertensive agents

In a randomized cross-over study of 12 healthy volunteers, RIF reduced the C_max_ by 39% and the AUC by 56% [115]. A placebo-controlled cross-over study in 9 healthy subjects found that RIF had a minor effect on the PK of atenolol, evidenced by a decrease of AUC_0-∞_ to 81% and an increase of renal clearance to 109% [116]. RIF decreased talinolol AUC by 21% after intravenous and by 35% after oral administration, probably through increased P-gp mediated excretion [117]. For teratrolol, a 35% decrease of teratrolol AUC was caused by RIF [118]. Single oral dose RIF reduced the mean oral bioavailability of nifedipine to 36%, decreased the t_1/2_, and increased the total clearance, but had no effect on t_max_ and apparent V_d_ [119].

##### Anti-epileptics

RIF significantly decreased lamotrigine exposure in a blinded study in 10 healthy volunteers, probably due to an induction of the UGT enzyme system [120].

##### Psychopharmaca

The AUC of bupropion dropped by 43% when co-administered with RIF, through induction of CYP2B6 [121]. Haloperidol and RIF co-administration led to a decrease in haloperidol AUC to 30% of baseline by day 28, whereas cessation of RIF in patients receiving haloperidol and RIF resulted in higher haloperidol AUCs up to 329% at day 28 [122]. In a two-phase open-label cross-over study, RIF decreased risperidone AUC by 72% and C_max_ by 50%, possibly through CYP3A4 induction [123]. A randomized placebo-controlled cross-over study of 6 healthy volunteers found the buspirone AUC to decrease by 91% and C_max_ by 85%, without a significant effect on the main active metabolite piperazine but with a 35% increase of piperazine C_max_ [124]. A similar effect on buspirone PK was observed in a different study, together with a significant reduction of effect of buspirone seen during 6 psychomotor tests, and attributed to a CYP3A4 interaction [125].

##### Anticoagulants

RIF enhanced clopidogrel-mediated platelet aggregation inhibition by 70%, possibly by induction of CYP3A4-mediated activation of clopidogrel [126]. RIF was found to significantly decrease prothrombin time and warfarin AUC [127].

##### Antihelmintic agents

Praziquantel was undetectable after a single dose of RIF in 7 of 10 subjects and in 5 of 10 after multi-dose administration, with the C_max_ decreasing by 99% and 98% respectively and the AUC by 94% and 89% [128]. In the other subjects the effect was less distinct.

##### Opioids

In a double-blinded randomized double-cross-over study of 10 healthy volunteers, RIF decreased the morphine AUC by 28% and the C_max_ by 41% as well as those of the morphine metabolites, resulting in a loss of analgesic effect [129]. RIF greatly reduced oral and intravenous oxycodon AUC by respectively 53% and 86% and reduced oral bioavailability from 69% to 21% through induction of CYP3A4, attenuating the PD effect of oxycodon [130].

##### Oncolytics

In a randomized cross-over study RIF reduced gefitinib AUC by 83% and C_max_ by 65% through induction of CYP3A4 [131]. RIF mediated CYP3A induction, reduced imatinib mesylate AUC_0–24h_ by 68%, AUC_0-∞_ by 74%, and C_max_ by 54%, and increased imatinib metabolite exposure [132].

##### Various

RIF increased atrasentan C_max_ by 150% and reduced t_1/2_ by 77%, but had no effect on AUC [133] and decreased the AUC of deferasirox, an oral iron chelator, by 44% [134]. Mirodenafil, used to treat erectile dysfunctions, has been studied in an open-label 1-sequence 3-period cross-over study of 19 healthy volunteers. It showed an RIF induced reduction of AUC of 97%, through CYP3A4 induction [135]. Prednisolon AUC was lower after co-treatment with RIF [136]. In a blinded 4-period placebo-controlled cross-over study, 8 healthy volunteers were administered RIF and pentoxifylline, resulting in a 35% increase of pentoxifylline AUC [137]. The effect of RIF on hydrodolasteron, the active metabolite of dolasteron, PK was small, *i.e.*, a reduction of AUC of 28% and a 17% reduction of C_max_ [138]. The concentration and t_1/2_ of theophylline decreased significantly after co-administration with RIF and the clearance increased by 53% within a week [139].

### 3.3. Macrolides

Macrolides are thought to exert their action by inhibition of bacterial protein synthesis, by preventing peptyltransfyrase-controlled attachement of a peptyl to tRNA to a following amino acid. Interactions of macrolides with enzyme systems and the effect of food on the PK of macrolides are briefly summarized in Table 3. The articles on which the summary has been based are further specified in the text below.

#### 3.3.1. Macrolides as Victims

##### Anti-retroviral drugs

AZM has been suggested to be a substrate for P-gp. A two-way open-label cross-over study of 12 healthy volunteers receiving AZM co-administered with nelfinavir, an inhibitor of P-gp, caused the AZM C_max_ and AUC to increase by more than 100% [140].

##### Antihistamines

A randomized open-label cross-over study of 24 healthy individuals found an interaction between AZM and the antiallergic agent rupatadine. Despite an alteration of the C_max_ of 3-hydroxydesloratadine (*i.e.*, metabolite of rupatadine), the PK parameters remained unchanged [141]. However, in a few studies a drug-drug interaction of AZM with antihistamines has been demonstrated. In a blinded randomized placebo-controlled study of 90 healthy patients receiving AZM and fexofenadine, the C_max_ and AUC increased by 69% and 67%, respectively, by an unknown mechanism [142]. Co-administration of cimetidine with CLR resulted in a decrease of the C_max_ of CLR and its metabolite 14-OH-CLR by 46% and 43% respectively. In addition, the t_max_ of 14-OH-CLR was prolonged from 1.39 to 2.34 hours (+68%) and the half life of both parent drug and metabolite increased, but neither AUC nor total clearance changed in this cross-over study (n = 12) [143].

##### Anti-inflammatory drugs

An open-label study of 66 patients in which patients were randomly assigned to one of three groups, concluded that co-administration of piroxicam and AZM interfered negatively with periodontal disposition, possibly by displacement of piroxicam of local acceptor sites at the level of periodontal tissues [144].

##### Food

The lack of effect of nutritional state on AZM tablets, as well as on sachets and pediatric suspension, was previously shown in an open-label cross-over study of 12 healthy volunteers [145]. The effect of food on AZM capsules and tablets has been studied in a four-way randomized cross-over study of 8 healthy volunteers [146]. The observations made in this study suggest that AZM capsules, in contrast to tablets, exhibit slow and/or delayed disintegration in the fed stomach. Prolonged gastric acid exposure caused an increased des-cladinose AZM (DCA) AUC of 243% in fasted state versus 270% in fed state. For CLR, a single dose cross-over design study showed that food intake directly before intake of a CLR tablet increased the extent of absorption by 25% and increased the AUC of the metabolite of CLR by approximately 9% [147]. An open-label cross-over study of 12 healthy volunteers showed that grapefruit juice, a well-known inhibitor of intestinal CYP3A4, had no effect on CLR PK parameters, except for an increased t_max_ [148]. For erythromycin (ERY) acistrate and enterocoated ERY bases the authors of a randomized cross-over study concluded that food intake had no effect on the PK of ERY acistrate and a slight effect on steady-state PK of the enterocoated ERY bases that might have some clinical relevance [149]. A single-phase open-label cross-over study in 18 healthy volunteers showed no effect of food intake on telitromycin PK [150].

##### Proton pump inhibitors

An open-label cross-over study of 23 *Helicobacter pylori*-positive patients and negative controls found exposure to CLR to decrease when co-administered with lanzoprazole, resulting in a C_max_ that decreased by respectively 40% and 15% and an AUC_0–10h_ by respectively 30% and 10% [151].

##### Various

No effect on PK parameters was observed when intravenous AZM and ceftriaxon were co-administered [152]. An absence of clinically relevant and/or statistically significant changes in PK was observed when AZM was co-administered with atovaquone [153], ceftriaxon [152], chloroquine [154], dideoxyinosine [155], RFB (also no PK interaction with CLR) [156], terfenadine [157], and zidovudine (only an increase in t_max_ of 44%) [155].

#### 3.3.2. Macrolides as Perpetrators

##### Anesthetics

A blinded randomized cross-over study of 10 healthy volunteers found an increase of the AUC of s-ketamine by 260% and the C_max_ by 360% after pre-treatment with oral CLR, probably by inhibition of its CYP3A metabolism [158]. The s-ketamine drug effect increased, but other behavioural effects and cold pain scores remained unaffected. Ropivacaine co-administration, studied in a double-blinded 3-way cross-over study in 8 healthy subjects, showed no changes in parent drug PK but a slight decrease in metabolite PK [159]. Administrating ERY with the local anesthetic lidocaine (lignocaine) has also been studied [160–162]. One study concluded that ERY either increases lidocaine metabolism to monoethylglycinexylidide (MEGX) or reduces further metabolism of MEGX, based on a 45–60% increase of MEGX AUC [162]. A subsequent study concluded that ERY increased the lidocaine AUC by 40–70% and the MEGX AUC by 40% [161].

##### Antiretroviral drugs

A comparative study showed that after combining the protease inhibitors tipranavir/ritonavir (TPV/r) with CLR the exposure of both TPV and CLR increased with 19% and 66%, respectively. This increase was moderately tolerated but careful monitoring for signs of toxicity, especially at higher doses of CLR was required [87]. This study suggests that CLR is not just an inhibitor of CYP3A4 but also act as a substrate. The effect of co-administering CLR and amprenavir, both substrates of CYP3A4 isoenzymes, has been studied in 12 subjects in an open-label randomized multiple-dose three-period crossover study. CLR increased the AUC, C_max_ and C_min_ of amprenavir by respectively 18%, 15% and 39%, albeit not of clinical importance [163]. In a open-label multi-dose cross-over study in 33 HIV patients, co-administration of ERY and saquinavir (soft gelatin capsules) resulted in a 99% increase of saquinavir AUC_0–8h_ [86].

Since antiretroviral drugs and macrolides are likely to be co-administered in patients with HIV and *Mycobacterium* infections, several studies focused on finding drug interactions between these two groups. An open-label randomized cross-over study in 22 patients focused on co-administration of CLR with ritonavir and found a small statistically significant increase of ritonavir exposure of approximately 10% [164]. A study with a similar design found a clinically non-significant effect of CLR on indinavir, *i.e.*, indinavir C_max_ increase of 58% and AUC of 47% in a smaller group of 12 patients at steady state [165]. Co-administering a darunavir/ritonavir combination with CLR in a randomized open-label cross-over study in 18 healthy volunteers resulted in a small increase of darunavir AUC by 13% and C_max_ by 17% [166]. During co-administration the CLR AUC increased by 57% and the C_max_ by 26%. In a RCT conducted on HIV patients AZM exhibited no effect on zidovudine PK, except for a decrease in t_max_ of 44%, suggesting both drugs can be co-administered safely [155]. A two-way open-label cross-over study of 12 healthy volunteers receiving AZM co-administered with nelfinavir caused the nelfinavir PK to decrease significantly, though not to be of clinical imporantance [140].

##### HMG-coA reductase inhibitors (statins)

CLR inhibits CYP3A4 mediated metabolism of several HMG-CoA reductase inhibitors such as atorvastatin, resulting in an increased AUC_24h_ of 82–400% [167,168]. Other drugs from the same group, such as pravastatin (a non-CYP substrate), showed an increase of almost 200%, and, for simvastatin, a 1000% increase of the AUC [168]. Simvastatin [169] and rosuvastatin [170], both used to treat hypercholesterolemia, were studied in combination with ERY. ERY increased the simvastatin AUC by 620% and C_max_ by 340%, whereas the rosuvastatin AUC decreased by 20% and C_max_ by 30%.

##### Proton pump inhibitors (PPIs)

Co-administration of CLR with proton pump inhibitors (PPIs), such as esomeprazole [171], lansoprazole [151,172–175], omeprazole [176–179], pantoprazole [176], and rabeprazole [180]—a combination commonly prescribed as part of *Helicobacter pylori* treatment—has been investigated in several studies. A double-blinded RCT in 18 healthy volunteers of different genotype groups of CYP2C19 found an increase of approximately 110% of the AUC and C_max_ of *(S)*-lansoprazole in extensive metabolizers (EMs) [172]. The increase was more pronounced in homozygous extensive metabolizers (homEMs) than in heterozygous extensive metabolizers (hetEMs). In poor metabolizers (PMs), CLR significantly increased the AUC and C_max_ of lansoprazole. The authors suggest the mechanism of the interaction was CYP3A4 inhibiton [172]. Another double-blinded RCT in 18 healthy Japanese volunteers found similar results [173]. Although the increase was slightly more pronounced in this study, the relative values between hetEMs, homEMs, and PMs were comparable. An 4-way cross-over RCT study in 12 *H. pylori*-negative volunteers looking into triple therapy consisting of lansoprazole, amoxicillin (AMX), and CLR compared to mono-therapy of each of the drugs found an increase of AUC of lansoprazole and 14-OH-CLR of 25% when co-administered as part of triple therapy [174]. No changes in AMX PK were found.

Omeprazole has also been widely studied in combination with CLR [176–178]. In a randomized double-blinded cross-over study of 20 healthy volunteers, CLR increased omeprazole AUC by 90% but did not result in a significantly altered gastric pH [178]. The study did find increased CLR and 14-OH-CLR levels in gastric tissue, *i.e.*, 16% in gastric fundus and approximately 900% in gastric mucus. Another double-blinded randomized cross-over study focused on CLR and omeprazole in 21 humans and found CLR to inhibit omeprazole metabolism, resulting in an increase of AUC of approximately 210% [177]. Also, a 200% increase was found in a randomized cross-over study co-administering esomeprazole and CLR to a group of 26 EMs and PMs [171]. A double-blinded cross-over study in 8 healthy volunteers not only studied co-administration of CLR with omeprazole, but also with pantoprazole, and found that the PK of CLR and pantoprazole remained unchanged. However, a twofold increase of omeprazole AUC and a decrease of omeprazole clearance and volume of distribution by respectively 75% and 56% was observed [176]. Rabeprazole, a proton pump inhibitor with minor CYP2C19 and 3A4 involvement, has been studied in a double-blinded RCT of 19 healthy subjects. When co-administered with CLR the AUC of rabeprazole did not change significantly, but the AUC of rabeprazole thioether was found to exhibit an approximate 200% increase [180]. Administration of roxithromycin with co-medication lansoprazole [181] or omeprazole [181] resulted in unchanged PK parameters.

##### Cardiovascular agents

In a prospective observational study combining CLR with digoxin, the digoxin clearance and elimination rate was 56–60% lower and the elimination half-life increased by 82% after co-administration, as was suggested by several other case reports [182]. An open-label Latin-square design study of nine healthy volunteers found that co-administration of intravenous digoxin with either CLR or ERY did not lead to increased AUCs, but did lead to prolonged Cl_renal_ by approximately 37% and urinary excretion of digoxin by 30%, for both CLR and ERY [183]. A study of 8 Japanese heart failure inpatients resulted in a dose-dependent increase of digoxin concentration by 70% at the common CLR dose of 400 mg per day [184]. A double-blinded cross-over study of 12 healthy volunteers showed that oral administration of CLR 250 mg twice a day resulted in a 70% increase of digoxin AUC, whereas intravenous administration of digoxin combined with CLR caused a mere 20% increase of digoxin AUC. The authors suggest the increased oral bioavailability and reduced renal clearance to be caused by P-gp inhibition [185]. When sotalol was co-administered with telithromycin the sotalol C_max_ decreased by 34% and AUC by 27%, leading to a decreased QTc interval by 15.5 ms [186]. Co-administration of ERY with talinolol resulted in a significant increase in talinolol AUC and C_max_ [187], and co-administration with felodipine resulted in an increase of felodipine AUC and C_max_ [188]. ERY was found to exhibit no effect on co-administered intravenous digoxine (except for a 1.4-fold increase of renal clearance) [183].

##### Antidiabetic drugs

In a randomized open-label cross-over study 12 healthy volunteers received glibenclamide and CLR, resulting in an increase of glibenclamide C_max_ to 125% and AUC to 135%, possibly through inhibition of P-gp mediated transport [189]. In a 2×2 double-blinded randomized study, nine healthy subjects received single doses of tolbutamide with or without CLR, resulting in a 20% increase of the tolbutamide absorption rate and a 26% increase of the mean bioavailability of tolbutamide [190]. As a result of simultaneous administration of tolbutamide and CLR, the authors reported a hypoglycemic effect. Co-administering ERY with glyburide resulted in an increase of glyburide C_max_ by 18% and a t_max_ decrease from 4.9 to 3.0 hours but with minimal effect on serum glucose concentration [191].

##### Antihistamines

A randomized open-label cross-over study focused on co-administration of loratadine with CLR in 24 healthy volunteers and found no PK changes for CLR. However, increases were observed of loratadine C_max_ by 36%, loratadine AUC of 76%, and increases in metabolite desloratadine AUC and C_max_, but no changes in QTc (<3%) [192]. The effect of AZM on older antihistamines has been researched in a randomized placebo-controlled study involving 18 volunteers. Small increases of less than 15% were found in desloratadine PK when co-administered with AZM, compared to an increase in fexofenadine C_max_ of 69% and AUC of 67% [142]. In a retrospective cohort study, co-administration of oral ERY with antihistamines, especially terfenadine, was associated with an increased risk of QTc prolongation [193]. Another antihistaminic agent, fexofenadine, showed an increase in AUC of 60% after co-administration with ERY and an increase in t_max_ to 3h (instead of 40 minutes) [194]. A lack of clinically relevant interaction was found between the antihistamine drug desloratadine and ERY, with an increase of desloratadine AUC of 10% and C_max_ of 20% [195]. When loratadine was co-administered with ERY, an increase in loratadine AUC of 40% was found, without clinically relevant changes in safety profile [196].

##### Benzodiazepines

Since some macrolides inhibit oxidative hepatic metabolism of various drugs, several studies have been conducted with the combination of AZM and midazolam [197–200]. A total of 64 healthy volunteers were included in a double-blinded randomized trial comparing the effect of co-administration of midazolam with AZM or ERY. The authors concluded that ERY, and not AZM, enhanced the objective and subjective effects of midazolam via interference of AZM with the hepatic CYP3A metabolism of midazolam [200]. A double-blind 2-phase cross-over study of 10 healthy volunteers contradicts these findings, as no changes in PD or PK of midazolam were found except for a decrease in C_max_ by 44% and a delay in t_max_ from 1.0 to 1.25 hours [199]. Two other open-label cross-over studies found no effect of AZM on midazolam PK or PD [197,198]. Co-administration of ERY with benzodiazepines has been investigated in several studies. For most benzodiazepines slight increases in AUC were measured, *i.e.*, alprazolam 61% [201], diazepam 15% [202], flunitrazepam 25% [202], nitrazepam 25% [203], triazolam by an uncalculated amount (C_max_ increased significantly) [204], and zopiclone 40% [205]. However, temazepam PK remained unchanged [206]. Midazolam exhibited the largest changes in AUC, ranging from 230–400% [207,208]. Administration of roxithromycin with midazolam resulted in a slight increase of midazolam AUC by 47% and t_1/2_ from 1.7 to 2.2 hours, accompanied by minor psychomotoric changes [209]. The authors suggest the midazolam PK changes to be caused by inhibition of CYP3A by roximycin. Intestinal and hepatic CYP3A inhibition by CLR resulted in a significant increase of midazolam AUC of 320% after intravenous administration and 800% after oral administration in an open-label comparative study of 16 healthy volunteers [210].

##### Immunosuppressants

A less recent study found that ERY increased the bioavailability of cyclosporin A from 36% to 60% by enhanced absorption [211]. The C_max_ and AUC of everolimus, a CYP3A4 substrate immunosuppressant, were increased by respectively 200% and 440% when co-administered with ERY [212]. An open-label placebo-controlled study of eight stable renal transplant patients found a 7% increase in AUC and 19% increase in C_max_ of cyclosporine A, a CYP3A4 substrate, after co-administration of AZM, and concluded the drug-drug interaction not to be clinically significant [213]. No effect on PK parameters was observed when spiramycin was combined with cyclosporine A [214].

##### Antithrombotics

A study without detailed described methodology found that ERY attenuates platelet aggregation inhibition caused by clopidogrel, leading to an increase of 73% in platelet aggregation [126]. The authors suggest the underlying mechanism to be enhanced activation of clopidogrel by ERY via CYP3A4. Co-administration of roxithromycin with warfarin resulted in unchanged PK parameters [215].

##### Bronchodilators

Combining CLR with theophylline in a Latin-square design study of 8 patients did not result in any significant PK changes [216]. Several less recent studies focused on the effect of ERY on theophylline PK and found an increased t_1/2_. Patients receiving ERY may therefore have an additional risk for the development of theophylline toxicity [217–220]. Co-administration of roxithromycin with theophylline resulted in unchanged PK parameters [221].

##### Glucocorticoids

A comparative study in 6 patients showed CLR to have no effect on prednisolon PK, but resulted in a 65% reduction of methylprednisolon clearance and significantly increased methylprednisolon concentrations [222].

##### Psychopharmaca

A double-dummy double-blinded cross-over study in 8 healthy subjects found the AUC of buspirone to increase by 600% and the C_max_ to increase by 500% [223]. ERY was found to exhibit no effect on the co-administered agent clozapine [224].

##### Anti-protozoic drugs

In a double-blinded cross-over study in 30 healthy volunteers co-administering ERY and quinine, a limited increase of total quinine levels was observed with disproportionately increased free quinine levels [225]. This was thought to be caused by irreversible inhibition of liver function-dependent CYP3A4-mediated metabolism and a displacement of quinine from plasma proteins. No escalated side effects or parasite clearance time was observed when spiramycin was combined with quinine [226]. A study of 24 patients concluded there is no PK interaction between AZM and chloroquine [154].

##### Anti-epileptics

When phenytoin was administered to patients also receiving josamycin, no PK changes were observed compared to monotherapy [217]. Finally, several studies found that ERY exhibited no effect on the co-administered agents oxcarbazepine [227], phenytoine [228], or tiagabin [229].

##### Opioids

Telithromycin combined with oxycodon in a blinded cross-over study resulted in an increased oxycodon AUC by 80% and a decreased AUC of the metabolite noroxycodon by 46% [230].

##### Antihelmintic drugs

A cross-over study of 18 volunteers found an increase of ivermectin AUC of 31% and C_max_ of 27% possibly by competition of AZM and ivermectin with P-gp, when combining AZM with albendazole and ivermectin [231].

##### Various

A comparative interaction study in 12 patients showed that CLR had no effect on dapsone PK [111], and a study without a clear study design of 12 HIV-positive patients found no differences in PK of 2′-3′-dideoxynosine either [129]. Cabergoline PK was studied in 10 healthy volunteers and 7 Parkinson patients after co-administration of CLR. This resulted in an increase of both the AUC and C_max_ of cabergoline by 260% in healthy volunteers and by 170% in Parkinson patients [232]. When ERY was co-administered with roflumilast, the AUC of the latter increased by 70% in an open-label single-dose comparative study of 16 healthy subjects [233]. Several studies found that ERY exhibited no effect on the co-administered agent, *i.e.*, acetaminophen [234], desmopressin (except for shortening t_max_) [235], or intranasal levocabastine [236]. AZM or CLR did not exhibit an effect on the PK parameters of atorvastatin either [167].

### 3.4. Fluoroquinolones

Fluoroquinolones exhibit their bactericidal effect through complex formation with bacterial DNA-gyrase, also known as topo-isomerase I, and with topo-isomerase IV. Interactions of the currently available fluoroquinolones (*i.e.*, ciprofloxacin [CIP], levofloxacin [LVX], gatifloxacin [GAT], MXF, and ofloxacin [OFX]) with enzyme systems and the effect of food on the PK of fluoroquinolones are briefly summarized in Table 4. The articles on which the summary is based are further specified in the text.

#### 3.4.1. Fluoroquinolones as Victims

##### Food

Several randomized cross-over studies have focused on bioavailability of fluoroquinolones when co-administered with food. Co-administration of a CIP suspension with a standard lunch had little effect on CIP PK parameters, reducing the C_max_ by 11% and increasing the AUC by 13% compared to a fasted state, in a randomized cross-over study of 68 healthy volunteers [237]. A comparable four-way cross-over study that included 6 healthy volunteers showed that intake with food had no effect on OFX PK parameters [238]. Enteral feeding reduced the C_max_ and AUC of CIP, depending on the method of administration, *i.e.*, orally (by 43% and 27% respectively), through gastrostomy tubes (by 37% and 53% respectively), or through jejunostomy (by 59% and 67% respectively) [239,240]. Both C_max_ and AUC of CIP were reduced by approximately 20% when co-administered with orange juice and by approximately 40% in case of concomitant treatment with calcium-fortified orange juice [241]. Dairy products, e.g., milk and yogurt, reduced the CIP exposure by 30% and 36% respectively when co-administered [242]. The OFX C_max_ and AUC were reduced by co-administration with enteral feeding by respectively 36% and 10% [240]. The relative bioavailability of GAT crushed tablet suspension was 99–109% when co-administered with enteral feeding in 16 patients [243]. Calcium-fortified orange juice slightly reduced the GAT C_max_ by 15% (non-significant) and the AUC by 12% [244], and a light morning meal appeared to have no effect on GAT C_max_ and reduced the AUC negligibly by 12% [245].

A high-fat breakfast combined with LVX in 24 healthy volunteers reduced the LVX C_max_ by 14% and the AUC by 10% [246]. However, in a randomized open-label 3-way cross-over study where LVX was co-administered with juice and cereal with and without milk, the AUC was reduced by approximately 15% [247]. Calcium-fortified orange juice reduced the LVX C_max_ by 18% without an effect on the AUC [248].

Crushed MXF could be administered with food via a nasogastric tube, resulting in a reduced C_max_ by 12% without effect on the AUC [249]. Co-administration of MXF together with dairy products is also possible, as yogurt reduced the C_max_ and AUC by 15% and 6% respectively [250].

##### Di- and trivalent metallic agents

Several studies focused on interactions between di- and trivalent metallic agents and fluoroquinolones. The proposed mechanism is that of a chelation complex formation with di- and trivalent metallic agents. When 600 mg iron (as gluconate) is co-administered with CIP in eight healthy volunteers, the C_max_ is reduced by 57% and the AUC by 67% [251]. In the same study, 300 mg iron (as sulphate) reduced these PK parameters by respectively 33% and 46%. A different study co-administering 100 mg ferrous sulphate with CIP or OFX found a decrease of C_max_ and AUC of respectively 54% and 57% for CIP, and of 36% and 25% for OFX [252]. Ferrous sulphate in a dose of 100 mg reduced the C_max_ of MXF by 59% and the AUC by 39% [253]. Also, lomefloxacin appeared to have an interaction with iron and resulted in reducing the C_max_ of lomefloxacin by 26% [254].

When Ca^2+^ was co-administered with CIP or gemifloxacin, fluoroquinolone exposure decreases and separated administration may be warranted as Ca^2+^ reduced CIP C_max_ and AUC by 48% [255], and reduced the gemifloxacin AUC by 21% and the C_max_ by 17% [256]. Remarkably, although the AUC remained unchanged, the C_max_ of LVX [257] and the third-generation fluoroquinolone MXF [258] decreased by approximately 18% by means of concomitant treatment with calcium. Probably a less stable chelating complex is formed in the latter case [258].

No clinical relevant changes in drug exposure—possibly through a less stable chelating complex—was found after administration of Al^3+^ with OFX in 10 healthy volunteers either [259]. A case control study suggested that di- and trivalent cation-containing compounds (DTCCs), including multivitamins, might inhibit fluoroquinolone absorption [260]. Also interactions with OTC preparations such as Centrum Forte^®^, containing Fe^2+^, Mg^2+^, Zn^2+^, Ca^2+^, Cu^2+^ and Mn^2+^, are observed, resulting in a decrease in C_max_ and AUC by 55% [251]. Finally, concomitant treatment with sevelamer hydrochloride, a phosphate-binding polymer, with CIP should be avoided as it reduced the CIP C_max_ by 40% and the AUC by 52% in 15 healthy volunteers [255]. This is also the case for concomitant treatment of CIP with lanthanum carbonate, which reduces both the CIP AUC and C_max_ by 55% [261].

##### Gastric acid-reducing agents

Maalox^®^, an antacid containing Mg^2+^ and Al^3+^, was combined with CIP [262], OFX [263], GAT [264], and MXF [265] in several studies, resulting in a clinically relevant reduction of absorption for all fluoroquinolones except for OFX. Maalox^®^ reduces CIP C_max_ after 5–10 min, 2 h and 4 h by 80%, 74%, and 13% respectively, and the CIP AUC by 85%, 77%, and 30% respectively [262]. GAT concomitantly administered with Maalox^®^ reduced the C_max_ by 68% and AUC by 64%, but when Maalox^®^ was administered 2h prior to GAT, the C_max_ was reduced by 45% and the AUC by 42% [264]. Co-administration of MXF with Maalox^®^ resulted in a reduced C_max_ when the antacid is administered simultaneously, 4h prior, or 2h after MXF, by respectively 61%, 1%, and 7%, and reduced AUC by 59%, 23%, and 26% at the aforementioned time points [265]. As an alternative for Maalox^®^, ranitidine is recommended. Co-administration of ranitidine with CIP [262] results in relatively unchanged C_max_ and AUC by 15%, and a combination with MXF [265] results in an increased C_max_ by 3% and an AUC by 8%. CIP treatment with concomitant omeprazole seems to be possible since a PK interaction is of non-significance for immediate release tablets [266] and extended-release tablets [267]. Sucralfate, containing Al^3+^, should not be concomitantly administered with CIP, LVX, OFX, or MXF. Four grams of sucralfate [268,269] reduced the AUC of CIP by 88–96% and 1 gram [270] by 30% when administered simultaneously. Sucralfate (1 g) also reduced the C_max_ and AUC of OFX [238,271] by respectively 70% and 61%, and of MXF [253] by respectively 71% and 60%, but did not affect LVX [246] PK parameters in a clinical relevant matter. Pepto Bismol^®^, containing Bi^3+^ and Al^3+^, did not reduce the drug exposure of CIP significantly, resulting in a decrease of C_max_ by 14% and AUC by 12% [272].

##### Antiretroviral drugs

The antiretroviral drug didanosine is unstable in an acidic environment and is kept stable by incorporating a buffer containing dihydroxylaluminium sodium carbonate and magnesium hydroxide in the formulation. These excipients interact with CIP and consequently result in a reduced C_max_ of CIP by 16–93% and AUC by 26–98% [273,274]. A new enteric bead formulation of didanosine allows concomitant administration with CIP, resulting in a reduced C_max_ and AUC of approximately 9% [274,275].

##### Anti-TB drugs

RFP modestly reduced MXF exposure by 17% and reduced the elimination half-life from 11.1 to 8.9 hours, possibly by inducing phase-II enzymes [43]. However, a PK interaction between RIF and MXF was observed in two prospective cohort studies [106,107]. RIF decreased MXF exposure by approximately 30%, possibly through induction of P-gp [107] combined with phase-II sulphatation and glucuronidation [106].

##### Anti-protozoic drugs

Co-administration of chloroquine with CIP increased the cumulative urinary concentration and excretion rate of CIP [276] and reduced the C_max_ by 22% [277] in five healthy male volunteers.

##### Analgesic

When CIP is co-administered with oral phenazopyridine, the CIP AUC increased by 30% and the t_max_ increased from 1h to 1.5h, but the C_max_ remained unchanged [278]. Competition for glomular filtration is a proposed mechanism.

##### Cardiaca

LVX has been suggested to inhibit metabolism of digoxin by intestinal bacteria to inactive metabolites which are subsequently excreted in urine and faeces, based on its broad antibacterial activity [279,280]. However, a clinically relevant interaction between digoxin and LVX was not observed [279]. For the newer fluoroquinolones sparfloxacin [281] and gemifloxacin [282] no clinically relevant interaction was observed with concomitant digoxin administration either.

##### Antigout drugs

Probenecid may possibly compete with fluoroquinolones for tubular secretion. Co-administration with CIP [283,284] and gemifloxacin [285] resulted in an increased drug exposure. Although the affinity for the renal transporter is greater for CIP and gemifloxacin than for probenecid, drug levels more than 100 times higher are observed for CIP and gemifloxacin.

#### 3.4.2. Fluoroquinolones as Perpetrators

##### Psychopharmaca

Several fluoroquinolones appear to inhibit CYP1A2, albeit to different extents. Drugs undergoing biotransformation through CYP1A2, such as caffeine by 3-N-demethylation, are at risk of interacting with fluoroquinolones [286]. Caffeine drug exposure has been increased by CIP, with an increase in C_max_ varying between 7–17% and an AUC increase ranging from 17 to 58%, depending on the CIP dose (200–1000 mg) in a prospective cohort study (n = 12) [287]. The same study found that OFX had negligible influence on caffeine PK parameters. Another study observed an increase in AUC and terminal phase half-life of caffeine by 145% and 115% respectively when co-administered with 1500 mg CIP [288]. The authors recommended that patients receiving CIP restrict their intake of caffeine. Of the newer fluoroquinolones, co-administration of clinafloxacin appeared to reduce caffeine clearance by 84%. As a result, the authors advised patients receiving clinafloxacin to restrict their caffeine intake [289]. Lomefloxacin, however, appeared not to have a significant effect on the caffeine disposition of 16 young healthy volunteers [290].

CIP was observed to induce clozapine and its metabolite *N*-desmethylclozapine serum concentration through CYP1A2 inhibition by approximately 30% in a randomized double-blinded cross-over study of 7 patients [291]. Several case reports suggest CIP exhibits a drug interaction with olanzapine, probably through CYP1A2 inhibition, resulting in QT prolongation [292,293].

##### Bronchodilators

Theophylline is also a CYP1A2 substrate. Therefore, patients receiving this agent are at risk for a drug-drug interation of theophylline with several fluoroquinolones. CIP, in a dose of 1000 mg, reduced theofylline clearance by 19–32% [216,294–298], whereas 600mg OFX co-administrated with caffeine led to unchanged theophylline disposition [299]. Co-administration of GAT [300] and MXF [301] with theophylline did not result in clinically relevant changes in PK parameters either. Of the newer fluoroquinolones, clinafloxacin or grepafloxacin changed theophylline PK parameters. Grepafloxacin (600 mg) significantly increased theophylline AUC and C_max_, and reduced theophylline clearance by 50% in 12 healthy volunteers [302]. Clinafloxacin reduced theophylline clearance dose-dependently by 50% (200 mg clinafloxacin) and 70% (400 mg clinafloxacin) [289]. However, lomefloxacin, gemifloxacin, sparfloxacin, and trovafloxacin appeared to have minimal effect on PK parameters of theophylline [298,303–305].

##### Anesthetics

CIP appeared to exhibit an interaction with anesthetics ropivacaine and lidocaine as well by means of CYP1A2 inhibition, resulting in an increased anesthetic exposure. CIP increased lidocaine AUC by 26% and C_max_ by 12% in a randomized double-blinded cross-over study of 9 patients [306]. In a similar study, CIP was found to reduce the clearance by 31%, without a significant effect on ropivacaine AUC and C_max_ [307]. LVX [308] and OFX increased the drug exposure of procainamide, probably through competition for tubular secretion through the organic cation transporter, resulting in significantly decreased renal clearance. When CIP was co-administered with procainamide, only the renal clearance of procainamide and its metabolite N-acetylprocainamide decreased significantly, leaving the other PK parameters unchanged [308].

##### Muscle relaxants

CIP was found to greatly increase the AUC and C_max_ of tizanide, a centrally acting muscle relaxant that is metabolized mainly by CYP1A2, by 874% and 583% respectively [309]. Because of escalated hypotensive and sedative effects of tizanide, physicians should avoid concomitant administration.

##### Immunosuppressants

Despite CYP3A4 involvement in the metabolism of cyclosporine, when CIP [310], MXF [311], or LVX [312], are co-administered with cyclosporine, no significant changes in cyclosporine exposure are observed in healthy volunteers [310,312] or patients [311]. The PK parameters of tacrolimus also remained unaffected after co-administration with MXF in a prospective cohort study that included 11 patients [311]. Inhibition of CYP3A4 by FQs is therefore unlikely.

##### Anti-diabetic drugs

Cases are reported of co-administration of fluoroquinolones, e.g., CIP and LVX, with different anti-diabetic agents, resulting in (refractory) diabetes, but the potential role of CYP3A4 is still uncertain and requires further research [313–315].

##### Anti-epileptic drugs

Clinafloxacin lowered phenytoin clearance by 15% when co-administered, possibly through CYP2C19 inhibition, leaving the clinafloxacin PK parameters unchanged [289].

##### Opioids

Several cases were reported of CIP co-administration with methadone resulting in QT prolongation, with a suggested mechanism being CIP inhibiting the CYP1A2 and 3A4-mediated metabolism of methadone [316–318].

##### Antimicrobial drugs

CIP did not have a clinically relevant interaction with RIF, with co-administration resulting in a decrease of RIF C_max_ by 12% without significant changes in AUC [319].

##### Anticoagulants

Co-administration of CIP with ximelagatran, an oral direct thrombin inhibitor that is a substrate for P-gp and is not metabolized by the CYP450 enzyme system, resulted in unchanged ximelagatran PK parameters [320].

### 3.5. β-Lactams

Although β-lactams are a relatively old group of antimicrobial drugs, only a few prospective drug interaction trials have been performed. Most of our knowledge on drug interactions is therefore based on case reports. These reports focus mainly on the CYP3A4 isoenzyme induction by flucloxacillin and nafcillin, the effect on the intestinal flora, the effect on (tubular) renal clearance of (co-)medication, induction of chemical degradation, and displacement of drugs from serum proteins.

A summary of the interactions is presented in Table 5. The data suggest that β-lactams have an effect on renal clearance of co-medication and vice versa. A possible underlying mechanism is the competitive character of this interaction, e.g., the drug with the highest affinity for the renal transporter is responsible for reducing renal tubular excretion of the competing drug.

#### 3.5.1. β-Lactams as Victims

##### Antimicrobial drugs

Probenecid has been reported to prolong the half-life and increase serum levels of cephalosporins cephradine and cefaclor [321]. Another study showed that probenecid prolonged the cefuroxim serum half-life by 63% in 10 healthy volunteers, probably by blocking tubular excretion. The beneficial effect is that the time that the concentration of cefuroxim is above the MIC90 of the causative pathogen was extended [322]. A recent prospective, non-randomized study examined the effect of probenecid on blocking tubular excretion of cefazolin. This study showed that probenecid 500 mg given orally 4 times a day enabled a cefazolin dose reduction to 2000 mg once a day, instead of 3 times a day, still resulting in therapeutic serum concentrations of cefazolin at steady state [323].

##### Electrolytes

In 2007, a FDA Medwatch alert described a decreased ceftriaxone recovery at higher calcium concentrations in two *in vitro* experiments carried out in blood subtracted from adult patients and umbilical cords. This might be due to formation of a ceftriaxone-calcium complex. The motivation for these *in vitro* studies and the following FDA Medwatch alert was a French case report presenting a case of an anaphylactic shock and calcium-ceftriaxone precipitation in a premature newborn.

#### 3.5.2. β-Lactams as Perpetrators

##### Oral anti-coagulants

Several case reports showed a decrease of the international normalized ratio (INR) when nafcillin or flucloxacillin is added to warfarin therapy [324–328]. This is suggested to be caused by an induction of hepatic CYP3A4 isoenzyme, resulting in an increased metabolism of warfarin.

AMX, with or without clavulanic acid, has been reported to increase the INR when combined with anticoagulant agents such as warfarin and acenocoumarol, possibly by reducing vitamin K-producing gut flora resulting in vitamin K deficiency [329,330]. An open-label case-control study including 302 cases concluded that penicillins were associated with a risk of overanticoagulation with an adjusted odds ratio of 24.2 (95% confidence interval). Amoxicillin plus clavulanic acid (AMC) should be avoided in patients receiving coumarins, or extra monitoring of the INR is recommended. However, a systematic prospective evaluation of the interaction between AMC and warfarin in 12 patients found that the INR was not modified in patients stable on warfarin therapy, in the absence of any infectious or inflammatory syndrome [331].

##### Calcium antagonist

A blinded randomized cross-over study of 9 healthy volunteers suggested an effect of nafcillin on CYP3A4. When nafcillin is combined with nifedipine, a substrate of CYP3A4, the AUC of nifedipine is significantly reduced by 63% and the total plasma clearance increased by 245% [332]. Another report warned not to combine verapamil with highly protein-bound drugs such as ceftriaxone, since these may displace verapamil from its protein-binding sites leading to verapamil toxicity [333].

##### Methotrexate (MTX)

Case reports described AMX to decrease renal tubular excretion, resulting in increased MTX concentrations [334]. The pencillin, oxacillin, has also been reported to increase MTX toxicity by reducing renal MTX clearance [335]. Piperacillin showed a similar interaction with drugs that are cleared renally, such as MTX [336]. A randomized open-label cross-over study of 10 healthy volunteers describes piperacillin to reduce renal and non-renal clearance of flucloxacillin by respectively 55% and 34% [337]. A proposed underlying mechanism was the higher affinity of piperacillin compared to other drugs, such as flucloxacillin or MTX, for the renal transporter responsible for renal tubular excretion resulting in competitive inhibition.

##### Antimicrobial drugs

Piperacillin has been shown to reduce half life and increase clearance of aminoglycosides in several studies by means of chemical degradation, a reaction believed to be dependent on many factors like temperature, contact time, concentration, and combined agents. A nucleophilic attack of the β-lactam ring of piperacillin on an amino group of the aminoglycoside seemed to be responsible for the drug-drug interaction [338–340].

Concomitant administration of mezlocillin with cefotaxime in eight healthy volunteers and five patients with end-stage renal disease (ESRD) suggested that the lower cefotaxime doses may prove to be adequate in patients with normal renal function with co-administered mezlocillin [341]. Although the mechanism of this nonrenal PK interaction is still unclear, the authors suspected the character of the interaction to be of metabolic origin [341].

##### Anti-epileptics

Seven case reports described a decreased effect of valproic acid when combined with meropenem, resulting in sub-therapeutic valproic acid levels and associated seizures [342–348]. Although the mechanism is not fully elucidated, two mechanisms were postulated: firstly the increased amount of cofactor uridine diphosphate glucuronic acid (UDPGA), increasing the glucuronidation in the liver, and secondly suppression of hydrolysis of the glucuronidated valproic acid in the liver [342]. Finally, after 60 hours of oxacillin treatment, the fraction of unbound phenytoin in a hypoalbuminemic patient rose insignificantly due to increased phenytoin displacement by oxacillin from the plasma protein-binding site [349].

##### Psychopharmaca

In three patients with preexisting or impending hypotension, imipenem has been reported to cause an episode of severe transient hypotension when combined with haloperidol. Competition of both drugs for the protein-binding site is one of the possible mechanisms responsible for an increase in the amount of protein-unbound haloperidol [350]. Finally, a case report suggested that co-administration of AMC with venlafaxine may result in serotonic toxicity [351].

##### Various

Imipenem was also described to have an interaction with theophyllin, resulting in drug-induced seizures in a series of 3 cases [352]. Combining cephalosporins and alcohol was discouraged too, since peripheral flush with shock was noticed [353]. Finally, a double-blinded randomized single-dose cross-over study of 12 healthy volunteers showed that co-administration of cephalexin altered the systemic disposition of metformin [354]. The authors concluded that cephalexin inhibits renal tubular secretion, increasing the metformin C_max_ by 34% and the AUC by 24%.

## 4. Conclusions

This article, presenting an overview of PK studies on drug-drug and drug-food interactions of macrolides, fluoroquinolones, rifamycines, oxazolidinones, and β-lactam antimicrobial drugs, can be used by physicians and pharmacists in daily practice to assist in preventing and managing PK drug interactions of antimicrobial drugs. The interactions presented in this article vary in extent of severity and clinical relevance. Potential clinical problems can range from therapeutic failure due to low drug exposure to adverse events due to toxic drug concentrations. PK interaction studies in both patients and healthy volunteers are included in this article. It has been demonstrated that PK characteristics of drugs can differ between healthy volunteers and patients [355]. As a result of an underlying disease, physiological changes can influence drug PK, although the mechanism remains to be elucidated. In many critically ill patients extracellular fluids have increased, possibly resulting in a higher volume of distribution that might affect PK [356]. One should bear in mind that findings in PK interaction studies performed in healthy volunteers might not be observed in clinical practice in specific patient populations.

Furthermore, PK interaction studies administering both single doses and multiple doses to study subjects were used in this overview. It need hardly be mentioned that multiple-dose studies will reflect best clinical practice. This is particularly true for PK interaction studies with biotransformation as possible underlying mechanism since induction of enzyme systems might require days to 2–3 weeks to develop fully [357]. The interaction may also persist at a similar length of time when the inducing agent is stopped. Unlike induction, inhibition of enzyme systems can occur within 2–3 days [357].

Physicians and pharmacists should also be aware of the fact that some of the included studies used doses that are higher or lower than those used in daily clinical practice. Especially in non-linear PK, this makes PK interactions difficult to interpret.

Finally, drug interactions not only occur when two or more interacting drugs are administered, but can also surface when one of the interacting drugs is halted. Most electronic health record systems include a program that can routinely check for drug-drug interactions and could assist in preventing drug interaction-related adverse events. However, these programs rarely check for interactions that can occur when one of the interacting drugs is halted. Multidisciplinary vigilance of physicians, pharmacists, and other health care professionals remains necessary for adequate management of drug-interactions of antimicrobial drugs.

## Figures and Tables

**Table 1.** Summary of interactions of the oxazolidinone LZD with enzyme systems and/or food.

	**Absorption Fat meal**	**Antacids**	**Metabolism: CYP**	**Excretion: P-gp**	**Reactive Oxygen Species**
**LZD**	↓	=	-	S*2	=

*1 Arrows pointing downward (↓) indicate inhibition and upward (↑) induction. The number of arrows indicates the extent of the inhibition or induction: 1 arrow <50%, 2 arrows 50–150%, 3 arrows >150% increase/decrease of AUC. “S” indicates the drug being a substrate, and “=” interaction is not relevant.

*2 Mostly based on case reports: in need of further research.

Note: Systematic research on newer compounds such as PNU-100480, posilozid (AZD2563), radezolid (RX-1741), torezolid, and others is not available. Since there were no interactions affecting displacement/distribution this process was not included in the table.

**Table 2.** Summary of interactions of rifamycines with enzyme systems and/or food.

	Rifamycines *1			
	**RIF**	**RFB**	**RFP**	**Rifalazil, rifaximin**
**Absorption:**				
Fat meal	↓	-	-	↓-↓↓
Antacids	0	-	-	-
OATP	↓↓-↑	-	-	-
**Metabolism:**	-			
CYP1A2	↑-↑↑	-	-	-
CYP2B6	↑	-	-	-
CYP2C8	↑-↑↑	-	-	-
CYP2C9	↑-↑↑	-	-	-
CYP2J2	↑	-	-	-
CYP3A4	**S**, ↑-↑↑↑	**S**, ↑-↑↑↑	-	0
UGT1A1	↑-↑↑	0-↑	-	-
**Excretion:**				
P-gp	↑-↑↑	-	-	-

*1 Arrows pointing downward (↓) indicate inhibition and upward (↑) induction. The number of arrows indicates the extent of the inhibition or induction: 1 arrow < 50%, 2 arrows 50–150%, 3 arrows >150% increase/decrease of AUC. “S” indicates the drug being a substrate, “0” indicates the absence of an interaction.

*2 Inhibition after single dose, upregulation after multiple doses.

Since there were no interactions affecting displacement/distribution this process was not included in the table.

**Table 3.** Summary of interactions of macrolides with enzyme systems and/or food.

	Macrolides *1				
	**Azithromycin**	**Clarithromycin**	**Erythromycin**	**Telithromycin**	**Roxithromycin**
**Absorption:**					
Food	0, ↓↓↓*2	↑	0	0	-
Antacids	-	↓	-	-	-
Grapefruit juice	-	0*3	-	-	-
P-gp	-	↓↓	-	-	-
**Metabolism:**	-	-	-	-	-
CYP2C19	-	↓↓↓	-	-	-
CYP3A4	0	**S**, ↓-↓↓↓	↓-↓↓↓	↓↓	↓
**Excretion:**					
P-gp	↓-↓↓	-	-	-	-

*1 Arrows pointing downward (↓) indicate inhibition and upward (↑) induction. The number of arrows indicates the extent of the inhibition or induction: 1 arrow < 50%, 2 arrows 50–150%, 3 arrows >150% increase/decrease of AUC. “S” indicates the drug being a substrate, “0” indicates the absence of an interaction.

*2 Absorption of capsules has been reduced, but absorption of tablets was unaffected by food.

*3 No effect on clarithromycin PK, except for an increase in t_max_.

Since there were no interactions affecting displacement/distribution this process was not included in the table.

**Table 4.** Summary of interactions of fluoroquinolones with enzyme systems and/or food.

	Fluoroquinolones *1							
	**Ciprofloxacin**	**Levofloxacin**	**Gatifloxacin**	**Moxifloxacin**	**Ofloxacin**	**Clinafloxacin**	**Lomefloxacin**	**Grepafloxacin**
**Absorption:**								
Meal	0-↓	↓	↓	↓	0	-	-	-
Enteral feeding	↓-↓↓*2		0	-	↓	-	-	-
Orange juice	↓	↓	-	-	-	-	-	-
(calcium-fortified)	(↓)	(↓)	(↓)					
Dairy products	↓	↓	-	0-↓	-	-	-	-
Di-/trivalent metallic agents	↓-↓↓	0-↓	-	0-↓	↓-↓↓	-	-	-
**Metabolism:**	-	-	-	-	-	-	-	-
Phase II enzymes	-	-	-	**S**	-	-	-	-
CYP1A2	↓-↓↓↓	-	0	0	0	↓↓	0	↓↓
CYP3A4	0	0	-	0	-	-	-	-
**Excretion:**								
Competitive tubular filtration	#	#	#	-	-	-	-	-
P-gp	0	-	-	-	-	-	-	-

*1 Arrows pointing downward (↓) indicate inhibition and upward (↑) induction. The number of arrows indicates the extent of the inhibition or induction: 1 arrow <50%, 2 arrows 50–150%, 3 arrows >150% increase/decrease of AUC. “S” indicates the drug being a substrate, “0” indicates the absence of an interaction, an “#” indicates the presence of an interaction.

*2 Depending on method of administration, *i.e.*, oral, through jejunostomy or through gastrostomy tubes.

Since there were no interactions affecting displacement/distribution this process was not included in the table.

**Table 5.** Summary of drug interactions of β-lactams.

β-lactams *1,^2^						
	**Nafcillin/flucloxacillin**	**Cephalexin, oxacillin, AMX**	**Piperacillin**	**Ceftriaxon**	**Imipenem, Meropenem**	Other *3
**Absorption:**	-	-	-	-	-	-
**Displacement/distribution:**						
Precipitation with Ca^2+^	-	-	-	#	-	-
Competitive binding on protein sites	-	-	-	↓	-	-
**Metabolism:**						
CYP3A4	↑↑-↑↑↑	-	-	-	-	-
Chemical degradation of aminoglycosids	-	-	↑	-	-	-
**Excretion:**						
Competitive tubular excretion	#	#	#	-	-	#

*1 Arrows pointing downward (↓) indicate inhibition and upward (↑) induction. The number of arrows indicates the extent of the interaction: 1 arrow <50%, 2 arrows 50–150%, 3 arrows >150%. “#” indicates the presence of an interaction.

*2 Mostly based on case reports: in need of further research.

*3 Cefaclor, cephalexin, cefuroxime, cefazolin, and cephradine.

## References

[1] Bruggemann R.J.M., Alffenaar J.W., Blijlevens N.M.A., Billaud E.M., Kosterink J.G., Verweij P.E., Burger D.M. (2008). Pharmacokinetic drug interactions of azoles. Curr. Fungal Infect. Rep..

[2] Egle H., Trittler R., Kummerer K., Lemmen S.W. (2005). Linezolid and rifampin: Drug interaction contrary to expectations?. Clin. Pharmacol. Ther..

[3] Wynalda M.A., Hauer M.J., Wienkers L.C. (2000). Oxidation of the novel oxazolidinone antibiotic linezolid in human liver microsomes. Drug Metab. Dispos..

[4] Bolhuis M.S., van Altena R., Uges D.R., van der Werf T.S., Kosterink J.G., Alffenaar J.W. (2010). Clarithromycin significantly increases linezolid serum concentrations. Antimicrob. Agents Chemother..

[5] Sisson T.L., Jungbluth G.L., Hopkins N.K. (1999). A pharmacokinetic evaluation of concomitant administration of linezolid and aztreonam. J. Clin. Pharmacol..

[6] Welshman I.R., Sisson T.A., Jungbluth G.L., Stalker D.J., Hopkins N.K. (2001). Linezolid absolute bioavailability and the effect of food on oral bioavailability. Biopharm. Drug Dispos..

[7] Gordi T., Tan L.H., Hong C., Hopkins N.J., Francom S.F., Slatter J.G., Antal E.J. (2003). The pharmacokinetics of linezolid are not affected by concomitant intake of the antioxidant vitamins C and E. J. Clin. Pharmacol..

[8] Grunder G., Zysset-Aschmann Y., Vollenweider F., Maier T., Krahenbuhl S., Drewe J. (2006). Lack of pharmacokinetic interaction between linezolid and antacid in healthy volunteers. Antimicrob. Agents Chemother..

[9] Hendershot P.E., Antal E.J., Welshman I.R., Batts D.H., Hopkins N.K. (2001). Linezolid: pharmacokinetic and pharmacodynamic evaluation of coadministration with pseudoephedrine HCl, phenylpropanolamine HCl, and dextromethorpan HBr. J. Clin. Pharmacol..

[10] Mason L.W., Randhawa K.S., Carpenter E.C. (2008). Serotonin toxicity as a consequence of linezolid use in revision hip arthroplasty. Orthopedics.

[11] Das P.K., Warkentin D.I., Hewko R., Forrest D.L. (2008). Serotonin syndrome after concomitant treatment with linezolid and meperidine. Clin. Infect. Dis..

[12] Packer S., Berman S.A. (2007). Serotonin syndrome precipitated by the monoamine oxidase inhibitor linezolid. Am. J. Psychiatry.

[13] Steinberg M., Morin A.K. (2007). Mild serotonin syndrome associated with concurrent linezolid and fluoxetine. Am. J. Health Syst. Pharm..

[14] Strouse T.B., Kerrihard T.N., Forscher C.A., Zakowski P. (2006). Serotonin syndrome precipitated by linezolid in a medically ill patient on duloxetine. J. Clin. Psychopharmacol..

[15] DeBellis R.J., Schaefer O.P., Liquori M., Volturo G.A. (2005). Linezolid-associated serotonin syndrome after concomitant treatment with citalopram and mirtazepine in a critically ill bone marrow transplant recipient. J. Intensive Care Med..

[16] Morales N., Vermette H. (2005). Serotonin syndrome associated with linezolid treatment after discontinuation of fluoxetine. Psychosomatics.

[17] Thomas C.R., Rosenberg M., Blythe V., Meyer W.J. (2004). Serotonin syndrome and linezolid. J. Am. Acad. Child Adolesc. Psychiatry.

[18] Jones S.L., Athan E., O'Brien D. (2004). Serotonin syndrome due to co-administration of linezolid and venlafaxine. J. Antimicrob. Chemother..

[19] Tahir N. (2004). Serotonin syndrome as a consequence of drug-resistant infections: an interaction between linezolid and citalopram. J. Am. Med. Dir. Assoc..

[20] Serio R.N. (2004). Acute delirium associated with combined diphenhydramine and linezolid use. Ann. Pharmacother..

[21] Hammerness P., Parada H., Abrams A. (2002). Linezolid: MAOI activity and potential drug interactions. Psychosomatics.

[22] Wigen C.L., Goetz M.B. (2002). Serotonin syndrome and linezolid. Clin. Infect. Dis..

[23] Lavery S., Ravi H., McDaniel W.W., Pushkin Y.R. (2001). Linezolid and serotonin syndrome. Psychosomatics.

[24] Aga V.M., Barklage N.E., Jefferson J.W. (2003). Linezolid, a monoamine oxidase inhibiting antibiotic, and antidepressants. J. Clin. Psychiatry.

[25] Polk R.E., Brophy D.F., Israel D.S., Patron R., Sadler B.M., Chittick G.E., Symonds W.T., Lou Y., Kristoff D., Stein D.S. (2001). Pharmacokinetic Interaction between amprenavir and rifabutin or rifampin in healthy males. Antimicrob. Agents Chemother..

[26] Jaruratanasirikul S., Sriwiriyajan S. (2001). Effect of indinavir on the pharmacokinetics of rifampicin in HIV-infected patients. J. Pharm. Pharmacol..

[27] Hamzeh F.M., Benson C., Gerber J., Currier J., McCrea J., Deutsch P., Ruan P., Wu H., Lee J., Flexner C. (2003). Steady-state pharmacokinetic interaction of modified-dose indinavir and rifabutin. Clin. Pharmacol. Ther..

[28] Kraft W.K., McCrea J.B., Winchell G.A., Carides A., Lowry R., Woolf E.J., Kusma S.E., Deutsch P.J., Greenberg H.E., Waldman S.A. (2004). Indinavir and rifabutin drug interactions in healthy volunteers. J. Clin. Pharmacol..

[29] Benator D.A., Weiner M.H., Burman W.J., Vernon A.A., Zhao Z.A., Khan A.E., Jones B.E., Sandman L., Engle M., Silva-Trigo C., Hsyu P.H., Becker M.I., Peloquin C.A. (2007). Clinical evaluation of the nelfinavir-rifabutin interaction in patients with tuberculosis and human immunodeficiency virus infection. Pharmacotherapy.

[30] Moyle G.J., Buss N.E., Goggin T., Snell P., Higgs C., Hawkins D.A. (2002). Interaction between saquinavir soft-gel and rifabutin in patients infected with HIV. Br. J. Clin. Pharmacol..

[31] Droste J.A., Verweij-van Wissen C.P., Kearney B.P., Buffels R., Vanhorssen P.J., Hekster Y.A., Burger D.M. (2005). Pharmacokinetic study of tenofovir disoproxil fumarate combined with rifampin in healthy volunteers. Antimicrob. Agents Chemother..

[32] Burger D.M., Agarwala S., Child M., Been-Tiktak A., Wang Y., Bertz R. (2006). Effect of rifampin on steady-state pharmacokinetics of atazanavir with ritonavir in healthy volunteers. Antimicrob. Agents Chemother..

[33] Li R.C., Narang P.K., Sahai J., Cameron W., Bianchine J.R. (1997). Rifabutin absorption in the gut unaltered by concomitant administration of didanosine in AIDS patients. Antimicrob. Agents Chemother..

[34] Peloquin C.A., Nitta A.T., Burman W.J., Brudney K.F., Miranda-Massari J.R., McGuinness M.E., Berning S.E., Gerena G.T. (1996). Low antituberculosis drug concentrations in patients with AIDS. Ann. Pharmacother..

[35] Jaruratanasirikul S., Kleepkaew A. (1996). Lack of effect of fluconazole on the pharmacokinetics of rifampicin in AIDS patients. J. Antimicrob. Chemother..

[36] Trapnell C.B., Narang P.K., Li R., Lavelle J.P. (1996). Increased plasma rifabutin levels with concomitant fluconazole therapy in HIV-infected patients. Ann. Intern. Med..

[37] Krishna G., Parsons A., Kantesaria B., Mant T. (2007). Evaluation of the pharmacokinetics of posaconazole and rifabutin following co-administration to healthy men. Curr. Med. Res. Opin..

[38] Alffenaar J.W., Nienhuis W.A., de Velde F., Zuur A.T., Wessels A.M., Almeida D., Grosset J., Adjei O., Uges D.R., van der Werf T.S. (2010). Pharmacokinetics of rifampin and clarithromycin in patients treated for Mycobacterium ulcerans infection. Antimicrob. Agents Chemother..

[39] Jordan M.K., Polis M.A., Kelly G., Narang P.K., Masur H., Piscitelli S.C. (2000). Effects of fluconazole and clarithromycin on rifabutin and 25-O-desacetylrifabutin pharmacokinetics. Antimicrob. Agents Chemother..

[40] Hafner R., Bethel J., Power M., Landry B., Banach M., Mole L., Standiford H.C., Follansbee S., Kumar P., Raasch R., Cohn D., Mushatt D., Drusano G. (1998). Tolerance and pharmacokinetic interactions of rifabutin and clarithromycin in human immunodeficiency virus-infected volunteers. Antimicrob. Agents Chemother..

[41] Bhatia R.S., Uppal R., Malhi R., Behera D., Jindal S.K. (1991). Drug interaction between rifampicin and cotrimoxazole in patients with tuberculosis. Hum. Exp. Toxicol..

[42] Shishoo C.J., Shah S.A., Rathod I.S., Savale S.S., Vora M.J. (2001). Impaired bioavailability of rifampicin in presence of isoniazid from fixed dose combination (FDC) formulation. Int. J. Pharm..

[43] Dooley K., Flexner C., Hackman J., Peloquin C.A., Nuermberger E., Chaisson R.E., Dorman S.E. (2008). Repeated administration of high-dose intermittent rifapentine reduces rifapentine and moxifloxacin plasma concentrations. Antimicrob. Agents Chemother..

[44] Orisakwe O.E., Akunyili D.N., Agbasi P.U., Ezejiofor N.A. (2004). Some plasma and saliva pharmacokinetics parameters of rifampicin in the presence of pefloxacin. Am. J. Ther..

[45] Jain A., Mehta V.L., Kulshrestha S. (1993). Effect of pyrazinamide on rifampicin kinetics in patients with tuberculosis. Tuber. Lung Dis..

[46] Peloquin C.A., Namdar R., Singleton M.D., Nix D.E. (1999). Pharmacokinetics of rifampin under fasting conditions, with food, and with antacids. Chest.

[47] Chen Y.X., Cabana B., Kivel N., Michaelis A. (2007). Effect of food on the pharmacokinetics of rifalazil, a novel antibacterial, in healthy male volunteers. J. Clin. Pharmacol..

[48] Park J.Y., Kim K.A., Park P.W., Park C.W., Shin J.G. (2003). Effect of rifampin on the pharmacokinetics and pharmacodynamics of gliclazide. Clin. Pharmacol. Ther..

[49] Niemi M., Kivisto K.T., Backman J.T., Neuvonen P.J. (2000). Effect of rifampicin on the pharmacokinetics and pharmacodynamics of glimepiride. Br. J. Clin. Pharmacol..

[50] Zheng H.X., Huang Y., Frassetto L.A., Benet L.Z. (2009). Elucidating rifampin's inducing and inhibiting effects on glyburide pharmacokinetics and blood glucose in healthy volunteers: unmasking the differential effects of enzyme induction and transporter inhibition for a drug and its primary metabolite. Clin. Pharmacol. Ther..

[51] Bidstrup T.B., Stilling N., Damkier P., Scharling B., Thomsen M.S., Brosen K. (2004). Rifampicin seems to act as both an inducer and an inhibitor of the metabolism of repaglinide. Eur. J. Clin. Pharmacol..

[52] Hatorp V., Hansen K.T., Thomsen M.S. (2003). Influence of drugs interacting with CYP3A4 on the pharmacokinetics, pharmacodynamics, and safety of the prandial glucose regulator repaglinide. J. Clin. Pharmacol..

[53] Niemi M., Backman J.T., Neuvonen M., Neuvonen P.J., Kivisto K.T. (2000). Rifampin decreases the plasma concentrations and effects of repaglinide. Clin. Pharmacol. Ther..

[54] Mitra S.K., Sundaram R., Venkataranganna M.V., Gopumadhavan S. (1999). Pharmacokinetic interaction of Diabecon (D-400) with rifampicin and nifedipine. Eur. J. Drug Metab Pharmacokinet..

[55] Hamman M.A., Bruce M.A., Haehner-Daniels B.D., Hall S.D. (2001). The effect of rifampin administration on the disposition of fexofenadine. Clin. Pharmacol. Ther..

[56] Shon J.H., Yeo C.W., Liu K.H., Lee S.S., Cha I.J., Shin J.G. (2010). Itraconazole and rifampin alter significantly the disposition and antihistamine effect of ebastine and its metabolites in healthy participants. J. Clin. Pharmacol..

[57] Backman J.T., Luurila H., Neuvonen M., Neuvonen P.J. (2005). Rifampin markedly decreases and gemfibrozil increases the plasma concentrations of atorvastatin and its metabolites. Clin. Pharmacol. Ther..

[58] Deng S., Chen X.P., Cao D., Yin T., Dai Z.Y., Luo J., Tang L., Li Y.J. (2009). Effects of a concomitant single oral dose of rifampicin on the pharmacokinetics of pravastatin in a two-phase, randomized, single-blind, placebo-controlled, crossover study in healthy Chinese male subjects. Clin. Ther..

[59] Kyrklund C., Backman J.T., Neuvonen M., Neuvonen P.J. (2004). Effect of rifampicin on pravastatin pharmacokinetics in healthy subjects. Br. J. Clin. Pharmacol..

[60] Zhang W., Deng S., Chen X.P., Zhou G., Xie H.T., He F.Y., Cao D., Li Y., Zhou H.H. (2008). Pharmacokinetics of rosuvastatin when coadministered with rifampicin in healthy males: a randomized, single-blind, placebo-controlled, crossover study. Clin. Ther..

[61] Kyrklund C., Backman J.T., Kivisto K.T., Neuvonen M., Laitila J., Neuvonen P.J. (2000). Rifampin greatly reduces plasma simvastatin and simvastatin acid concentrations. Clin. Pharmacol. Ther..

[62] Park J.Y., Kim K.A., Kang M.H., Kim S.L., Shin J.G. (2004). Effect of rifampin on the pharmacokinetics of rosiglitazone in healthy subjects. Clin. Pharmacol. Ther..

[63] Jaakkola T., Backman J.T., Neuvonen M., Laitila J., Neuvonen P.J. (2006). Effect of rifampicin on the pharmacokinetics of pioglitazone. Br. J. Clin. Pharmacol..

[64] Niemi M., Backman J.T., Neuvonen M., Neuvonen P.J. (2003). Effect of rifampicin on the pharmacokinetics and pharmacodynamics of nateglinide in healthy subjects. Br. J. Clin. Pharmacol..

[65] Kim Y.H., Yoon Y.R., Kim Y.W., Shin J.G., Cha I.J. (1998). Effects of rifampin on cyclosporine disposition in kidney recipients with tuberculosis. Transplant. Proc..

[66] Kovarik J.M., Hartmann S., Figueiredo J., Rouilly M., Port A., Rordorf C. (2002). Effect of rifampin on apparent clearance of everolimus. Ann. Pharmacother..

[67] Naesens M., Kuypers D.R., Streit F., Armstrong V.W., Oellerich M., Verbeke K., Vanrenterghem Y. (2006). Rifampin induces alterations in mycophenolic acid glucuronidation and elimination: implications for drug exposure in renal allograft recipients. Clin. Pharmacol. Ther..

[68] Hebert M.F., Fisher R.M., Marsh C.L., Dressler D., Bekersky I. (1999). Effects of rifampin on tacrolimus pharmacokinetics in healthy volunteers. J. Clin. Pharmacol..

[69] Boni J., Leister C., Burns J., Cincotta M., Hug B., Moore L. (2007). Pharmacokinetic profile of temsirolimus with concomitant administration of cytochrome p450-inducing medications. J. Clin. Pharmacol..

[70] Haas D.W., Koletar S.L., Laughlin L., Kendall M.A., Suckow C., Gerber J.G., Zolopa A.R., Bertz R., Child M.J., Hosey L., Alston-Smith B., Acosta E.P. (2009). Hepatotoxicity and gastrointestinal intolerance when healthy volunteers taking rifampin add twice-daily atazanavir and ritonavir. J. Acquir. Immune. Defic. Syndr..

[71] Sekar V., Lavreys L., Van de Casteele T., Berckmans C., Spinosa-Guzman S., Vangeneugden T., De Pauw M., Hoetelmans R. (2010). Pharmacokinetics of darunavir/ritonavir and rifabutin coadministered in HIV-negative healthy volunteers. Antimicrob. Agents Chemother..

[72] Borin M.T., Chambers J.H., Carel B.J., Gagnon S., Freimuth W.W. (1997). Pharmacokinetic study of the interaction between rifampin and delavirdine mesylate. Clin. Pharmacol. Ther..

[73] Borin M.T., Chambers J.H., Carel B.J., Freimuth W.W., Aksentijevich S., Piergies A.A. (1997). Pharmacokinetic study of the interaction between rifabutin and delavirdine mesylate in HIV-1 infected patients. Antiviral Res..

[74] Sahai J., Narang P.K., Hawley-Foss N., Li R.C., Kamal M., Cameron D.W. (1995). A phase I evaluation of concomitant rifabutin and didanosine in symptomatic HIV-infected patients. J. Acquir. Immune. Defic. Syndr. Hum. Retrovirol..

[75] Boyd M.A., Zhang X., Dorr A., Ruxrungtham K., Kolis S., Nieforth K., Kinchelow T., Buss N., Patel I.H. (2003). Lack of enzyme-inducing effect of rifampicin on the pharmacokinetics of enfuvirtide. J. Clin. Pharmacol..

[76] Lopez-Cortes L.F., Ruiz-Valderas R., Viciana P., Alarcon-Gonzalez A., Gomez-Mateos J., Leon-Jimenez E., Sarasanacenta M., Lopez-Pua Y., Pachon J. (2002). Pharmacokinetic interactions between efavirenz and rifampicin in HIV-infected patients with tuberculosis. Clin. Pharmacokinet..

[77] Matteelli A., Regazzi M., Villani P., De Iaco G., Cusato M., Carvalho A.C., Caligaris S., Tomasoni L., Manfrin M., Capone S., Carosi G. (2007). Multiple-dose pharmacokinetics of efavirenz with and without the use of rifampicin in HIV-positive patients. Curr. HIV. Res..

[78] Ren Y., Nuttall J.J., Eley B.S., Meyers T.M., Smith P.J., Maartens G., McIlleron H.M. (2009). Effect of rifampicin on efavirenz pharmacokinetics in HIV-infected children with tuberculosis. J. Acquir. Immune. Defic. Syndr..

[79] Ford S.L., Chen Y.C., Lou Y., Borland J., Min S.S., Yuen G.J., Shelton M.J. (2008). Pharmacokinetic interaction between fosamprenavir-ritonavir and rifabutin in healthy subjects. Antimicrob. Agents Chemother..

[80] Justesen U.S., Andersen A.B., Klitgaard N.A., Brosen K., Gerstoft J., Pedersen C. (2004). Pharmacokinetic interaction between rifampin and the combination of indinavir and low-dose ritonavir in HIV-infected patients. Clin. Infect. Dis..

[81] Nijland H.M., L'homme R.F., Rongen G.A., van Uden P., van Crevel R., Boeree M.J., Aarnoutse R.E., Koopmans P.P., Burger D.M. (2008). High incidence of adverse events in healthy volunteers receiving rifampicin and adjusted doses of lopinavir/ritonavir tablets. AIDS.

[82] Abel S., Jenkins T.M., Whitlock L.A., Ridgway C.E., Muirhead G.J. (2008). Effects of CYP3A4 inducers with and without CYP3A4 inhibitors on the pharmacokinetics of maraviroc in healthy volunteers. Br. J. Clin. Pharmacol..

[83] Cohen K., van Cutsem G., Boulle A., McIlleron H., Goemaere E., Smith P.J., Maartens G. (2008). Effect of rifampicin-based antitubercular therapy on nevirapine plasma concentrations in South African adults with HIV-associated tuberculosis. J. Antimicrob. Chemother..

[84] Elsherbiny D., Cohen K., Jansson B., Smith P., McIlleron H., Simonsson U.S. (2009). Population pharmacokinetics of nevirapine in combination with rifampicin-based short course chemotherapy in HIV- and tuberculosis-infected South African patients. Eur. J. Clin. Pharmacol..

[85] Wenning L.A., Hanley W.D., Brainard D.M., Petry A.S., Ghosh K., Jin B., Mangin E., Marbury T.C., Berg J.K., Chodakewitz J.A., Stone J.A., Gottesdiener K.M., Wagner J.A., Iwamoto M. (2009). Effect of rifampin, a potent inducer of drug-metabolizing enzymes, on the pharmacokinetics of raltegravir. Antimicrob. Agents Chemother..

[86] Grub S., Bryson H., Goggin T., Ludin E., Jorga K. (2001). The interaction of saquinavir (soft gelatin capsule) with ketoconazole, erythromycin and rifampicin: comparison of the effect in healthy volunteers and in HIV-infected patients. Eur. J. Clin. Pharmacol..

[87] la Porte C.J., Sabo J.P., Elgadi M., Cameron D.W. (2009). Interaction studies of tipranavir-ritonavir with clarithromycin, fluconazole, and rifabutin in healthy volunteers. Antimicrob. Agents Chemother..

[88] Backman J.T., Granfors M.T., Neuvonen P.J. (2006). Rifampicin is only a weak inducer of CYP1A2-mediated presystemic and systemic metabolism: studies with tizanidine and caffeine. Eur. J. Clin. Pharmacol..

[89] Li R.C., Nightingale S., Lewis R.C., Colborn D.C., Narang P.K. (1996). Lack of effect of concomitant zidovudine on rifabutin kinetics in patients with AIDS-related complex. Antimicrob. Agents Chemother..

[90] Gallicano K., Sahai J., Swick L., Seguin I., Pakuts A., Cameron D.W. (1995). Effect of rifabutin on the pharmacokinetics of zidovudine in patients infected with human immunodeficiency virus. Clin. Infect. Dis..

[91] Gallicano K.D., Sahai J., Shukla V.K., Seguin I., Pakuts A., Kwok D., Foster B.C., Cameron D.W. (1999). Induction of zidovudine glucuronidation and amination pathways by rifampicin in HIV-infected patients. Br. J. Clin. Pharmacol..

[92] Burger D.M., Meenhorst P.L., ten Napel C.H., Mulder J.W., Neef C., Koks C.H., Bult A., Beijnen J.H. (1994). Pharmacokinetic variability of zidovudine in HIV-infected individuals: subgroup analysis and drug interactions. AIDS.

[93] Burger D.M., Meenhorst P.L., Koks C.H., Beijnen J.H. (1993). Pharmacokinetic interaction between rifampin and zidovudine. Antimicrob. Agents Chemother..

[94] Pentikis H.S., Connolly M., Trapnell C.B., Forbes W.P., Bettenhausen D.K. (2007). The effect of multiple-dose, oral rifaximin on the pharmacokinetics of intravenous and oral midazolam in healthy volunteers. Pharmacotherapy.

[95] Backman J.T., Olkkola K.T., Neuvonen P.J. (1996). Rifampin drastically reduces plasma concentrations and effects of oral midazolam. Clin. Pharmacol. Ther..

[96] Backman J.T., Kivisto K.T., Olkkola K.T., Neuvonen P.J. (1998). The area under the plasma concentration-time curve for oral midazolam is 400-fold larger during treatment with itraconazole than with rifampicin. Eur. J. Clin. Pharmacol..

[97] Villikka K., Kivisto K.T., Backman J.T., Olkkola K.T., Neuvonen P.J. (1997). Triazolam is ineffective in patients taking rifampin. Clin. Pharmacol. Ther..

[98] Villikka K., Kivisto K.T., Luurila H., Neuvonen P.J. (1997). Rifampin reduces plasma concentrations and effects of zolpidem. Clin. Pharmacol. Ther..

[99] Villikka K., Kivisto K.T., Lamberg T.S., Kantola T., Neuvonen P.J. (1997). Concentrations and effects of zopiclone are greatly reduced by rifampicin. Br. J. Clin. Pharmacol..

[100] Stone J.A., Migoya E.M., Hickey L., Winchell G.A., Deutsch P.J., Ghosh K., Freeman A., Bi S., Desai R., Dilzer S.C., Lasseter K.C., Kraft W.K., Greenberg H., Waldman S.A. (2004). Potential for interactions between caspofungin and nelfinavir or rifampin. Antimicrob. Agents Chemother..

[101] Panomvana Na Ayudhya D., Thanompuangseree N., Tansuphaswadikul S. (2004). Effect of rifampicin on the pharmacokinetics of fluconazole in patients with AIDS. Clin. Pharmacokinet..

[102] Jaruratanasirikul S., Sriwiriyajan S. (1998). Effect of rifampicin on the pharmacokinetics of itraconazole in normal volunteers and AIDS patients. Eur. J. Clin. Pharmacol..

[103] Hafner R., Bethel J., Standiford H.C., Follansbee S., Cohn D.L., Polk R.E., Mole L., Raasch R., Kumar P., Mushatt D., Drusano G. (2001). Tolerance and pharmacokinetic interactions of rifabutin and azithromycin. Antimicrob. Agents Chemother..

[104] Ribera E., Pou L., Fernandez-Sola A., Campos F., Lopez R.M., Ocana I., Ruiz I., Pahissa A. (2001). Rifampin reduces concentrations of trimethoprim and sulfamethoxazole in serum in human immunodeficiency virus-infected patients. Antimicrob. Agents Chemother..

[105] Colmenero J.D., Fernandez-Gallardo L.C., Agundez J.A., Sedeno J., Benitez J., Valverde E. (1994). Possible implications of doxycycline-rifampin interaction for treatment of brucellosis. Antimicrob. Agents Chemother..

[106] Nijland H.M., Ruslami R., Suroto A.J., Burger D.M., Alisjahbana B., van Crevel R., Aarnoutse R.E. (2007). Rifampicin reduces plasma concentrations of moxifloxacin in patients with tuberculosis. Clin. Infect. Dis..

[107] Weiner M., Burman W., Luo C.C., Peloquin C.A., Engle M., Goldberg S., Agarwal V., Vernon A. (2007). Effects of rifampin and multidrug resistance gene polymorphism on concentrations of moxifloxacin. Antimicrob. Agents Chemother..

[108] Humbert G., Brumpt I., Montay G., Le Liboux A., Frydman A., Borsa-Lebas F., Moore N. (1991). Influence of rifampin on the pharmacokinetics of pefloxacin. Clin. Pharmacol. Ther..

[109] Wanwimolruk S., Kang W., Coville P.F., Viriyayudhakorn S., Thitiarchakul S. (1995). Marked enhancement by rifampicin and lack of effect of isoniazid on the elimination of quinine in man. Br. J. Clin. Pharmacol..

[110] Pukrittayakamee S., Prakongpan S., Wanwimolruk S., Clemens R., Looareesuwan S., White N.J. (2003). Adverse effect of rifampin on quinine efficacy in uncomplicated falciparum malaria. Antimicrob. Agents Chemother..

[111] Winter H.R., Trapnell C.B., Slattery J.T., Jacobson M., Greenspan D.L., Hooton T.M., Unadkat J.D. (2004). The effect of clarithromycin, fluconazole, and rifabutin on dapsone hydroxylamine formation in individuals with human immunodeficiency virus infection (AACTG 283). Clin. Pharmacol. Ther..

[112] Chen Y.X., Cabana B., Kivel N., Pieniaszek H., Gilman S., Michaelis A. (2007). Lack of effect of rifalazil on ethinyl estradiol pharmacokinetics in healthy postmenopausal women. Int. J. Clin. Pharmacol. Ther..

[113] Trapnell C.B., Connolly M., Pentikis H., Forbes W.P., Bettenhausen D.K. (2007). Absence of effect of oral rifaximin on the pharmacokinetics of ethinyl estradiol/norgestimate in healthy females. Ann. Pharmacother..

[114] Kivisto K.T., Villikka K., Nyman L., Anttila M., Neuvonen P.J. (1998). Tamoxifen and toremifene concentrations in plasma are greatly decreased by rifampin. Clin. Pharmacol. Ther..

[115] Tapaninen T., Neuvonen P.J., Niemi M. (2010). Rifampicin reduces the plasma concentrations and the renin-inhibiting effect of aliskiren. Eur. J. Clin. Pharmacol..

[116] Lilja J.J., Juntti-Patinen L., Neuvonen P.J. (2006). Effect of rifampicin on the pharmacokinetics of atenolol. Basic Clin. Pharmacol. Toxicol..

[117] Westphal K., Weinbrenner A., Zschiesche M., Franke G., Knoke M., Oertel R., Fritz P., von Richter O., Warzok R., Hachenberg T., Kauffmann H.M., Schrenk D., Terhaag B., Kroemer H.K., Siegmund W. (2000). Induction of P-glycoprotein by rifampin increases intestinal secretion of talinolol in human beings: a new type of drug/drug interaction. Clin. Pharmacol. Ther..

[118] Kirch W., Milferstadt S., Halabi A., Rocher I., Efthymiopoulos C., Jung L. (1990). Interaction of tertatolol with rifampicin and ranitidine pharmacokinetics and antihypertensive activity. Cardiovasc. Drugs Ther..

[119] Ndanusa B.U., Mustapha A., Abdu-Aguye I. (1997). The effect of single does of rifampicin on the pharmacokinetics of oral nifedipine. J. Pharm. Biomed. Anal..

[120] Ebert U., Thong N.Q., Oertel R., Kirch W. (2000). Effects of rifampicin and cimetidine on pharmacokinetics and pharmacodynamics of lamotrigine in healthy subjects. Eur. J. Clin. Pharmacol..

[121] Loboz K.K., Gross A.S., Williams K.M., Liauw W.S., Day R.O., Blievernicht J.K., Zanger U.M., McLachlan A.J. (2006). Cytochrome P450 2B6 activity as measured by bupropion hydroxylation: effect of induction by rifampin and ethnicity. Clin. Pharmacol. Ther..

[122] Kim Y.H., Cha I.J., Shim J.C., Shin J.G., Yoon Y.R., Kim Y.K., Kim J.I., Park G.H., Jang I.J., Woo J.I., Shin S.G. (1996). Effect of rifampin on the plasma concentration and the clinical effect of haloperidol concomitantly administered to schizophrenic patients. J. Clin. Psychopharmacol..

[123] Mahatthanatrakul W., Nontaput T., Ridtitid W., Wongnawa M., Sunbhanich M. (2007). Rifampin, a cytochrome P450 3A inducer, decreases plasma concentrations of antipsychotic risperidone in healthy volunteers. J. Clin. Pharm. Ther..

[124] Kivisto K.T., Lamberg T.S., Neuvonen P.J. (1999). Interactions of buspirone with itraconazole and rifampicin: effects on the pharmacokinetics of the active 1-(2-pyrimidinyl)-piperazine metabolite of buspirone. Pharmacol. Toxicol..

[125] Lamberg T.S., Kivisto K.T., Neuvonen P.J. (1998). Concentrations and effects of buspirone are considerably reduced by rifampicin. Br. J. Clin. Pharmacol..

[126] Lau W.C., Waskell L.A., Watkins P.B., Neer C.J., Horowitz K., Hopp A.S., Tait A.R., Carville D.G., Guyer K.E., Bates E.R. (2003). Atorvastatin reduces the ability of clopidogrel to inhibit platelet aggregation: a new drug-drug interaction. Circulation.

[127] O'Reilly R.A. (1974). Interaction of sodium warfarin and rifampin. Studies in man. Ann. Intern. Med..

[128] Ridtitid W., Wongnawa M., Mahatthanatrakul W., Punyo J., Sunbhanich M. (2002). Rifampin markedly decreases plasma concentrations of praziquantel in healthy volunteers. Clin. Pharmacol. Ther..

[129] Gillum J.G., Bruzzese V.L., Israel D.S., Kaplowitz L.G., Polk R.E. (1996). Effect of clarithromycin on the pharmacokinetics of 2′,3′-dideoxyinosine in patients who are seropositive for human immunodeficiency virus. Clin. Infect. Dis..

[130] Nieminen T.H., Hagelberg N.M., Saari T.I., Pertovaara A., Neuvonen M., Laine K., Neuvonen P.J., Olkkola K.T. (2009). Rifampin greatly reduces the plasma concentrations of intravenous and oral oxycodone. Anesthesiology.

[131] Swaisland H.C., Ranson M., Smith R.P., Leadbetter J., Laight A., McKillop D., Wild M.J. (2005). Pharmacokinetic drug interactions of gefitinib with rifampicin, itraconazole and metoprolol. Clin. Pharmacokinet..

[132] Bolton A.E., Peng B., Hubert M., Krebs-Brown A., Capdeville R., Keller U., Seiberling M. (2004). Effect of rifampicin on the pharmacokinetics of imatinib mesylate (Gleevec, STI571) in healthy subjects. Cancer Chemother. Pharmacol..

[133] Xiong H., Carr R.A., Locke C.S., Katz D.A., Achari R., Doan T.T., Wang P., Jankowski J.R., Sleep D.J. (2007). Dual effects of rifampin on the pharmacokinetics of atrasentan. J. Clin. Pharmacol..

[134] Skerjanec A., Wang J., Maren K., Rojkjaer L. (2010). Investigation of the pharmacokinetic interactions of deferasirox, a once-daily oral iron chelator, with midazolam, rifampin, and repaglinide in healthy volunteers. J. Clin. Pharmacol..

[135] Shin K.H., Kim B.H., Kim T.E., Kim J.W., Yi S., Yoon S.H., Cho J.Y., Shin S.G., Jang I.J., Yu K.S. (2009). The effects of ketoconazole and rifampicin on the pharmacokinetics of mirodenafil in healthy Korean male volunteers: an open-label, one-sequence, three-period, three-treatment crossover study. Clin. Ther..

[136] Lee K.H., Shin J.G., Chong W.S., Kim S., Lee J.S., Jang I.J., Shin S.G. (1993). Time course of the changes in prednisolone pharmacokinetics after co-administration or discontinuation of rifampin. Eur. J. Clin. Pharmacol..

[137] Magnusson M., Bergstrand I.C., Bjorkman S., Heijl A., Roth B., Hoglund P. (2006). A placebo-controlled study of retinal blood flow changes by pentoxifylline and metabolites in humans. Br. J. Clin. Pharmacol..

[138] Dimmitt D.C., Cramer M.B., Keung A., Arumugham T., Weir S.J. (1999). Pharmacokinetics of dolasetron with coadministration of cimetidine or rifampin in healthy subjects. Cancer Chemother. Pharmacol..

[139] Ahn H.C., Lee Y.C. (2003). The clearance of theophylline is increased during the initial period of tuberculosis treatment. Int. J. Tuberc. Lung Dis..

[140] Amsden G.W., Nafziger A.N., Foulds G., Cabelus L.J. (2000). A study of the pharmacokinetics of azithromycin and nelfinavir when coadministered in healthy volunteers. J. Clin. Pharmacol..

[141] Solans A., Izquierdo I., Donado E., Antonijoan R., Pena J., Nadal T., Carbo M.L., Merlos M., Barbanoj M. (2008). Pharmacokinetic and safety profile of rupatadine when coadministered with azithromycin at steady-state levels: a randomized, open-label, two-way, crossover, Phase I study. Clin. Ther..

[142] Gupta S., Banfield C., Kantesaria B., Marino M., Clement R., Affrime M., Batra V. (2001). Pharmacokinetic and safety profile of desloratadine and fexofenadine when coadministered with azithromycin: a randomized, placebo-controlled, parallel-group study. Clin. Ther..

[143] Amsden G.W., Cheng K.L., Peloquin C.A., Nafziger A.N. (1998). Oral cimetidine prolongs clarithromycin absorption. Antimicrob. Agents Chemother..

[144] Malizia T., Batoni G., Ghelardi E., Baschiera F., Graziani F., Blandizzi C., Gabriele M., Campa M., Del Tacca M., Senesi S. (2001). Interaction between piroxicam and azithromycin during distribution to human periodontal tissues. J. Periodontol..

[145] Foulds G., Luke D.R., Teng R., Willavize S.A., Friedman H., Curatolo W.J. (1996). The absence of an effect of food on the bioavailability of azithromycin administered as tablets, sachet or suspension. J. Antimicrob. Chemother..

[146] Curatolo W., Foulds G., Labadie R. (2010). Mechanistic study of the azithromycin dosage-form-dependent food effect. Pharm. Res..

[147] Chu S., Park Y., Locke C., Wilson D.S., Cavanaugh J.C. (1992). Drug-food interaction potential of clarithromycin, a new macrolide antimicrobial. J. Clin. Pharmacol..

[148] Cheng K.L., Nafziger A.N., Peloquin C.A., Amsden G.W. (1998). Effect of grapefruit juice on clarithromycin pharmacokinetics. Antimicrob. Agents Chemother..

[149] Tuominen R.K., Mannisto P.T., Pohto P., Solkinen A., Vuorela A. (1988). Absorption of erythromycin acistrate and erythromycin base in the fasting and non-fasting state. J. Antimicrob. Chemother..

[150] Bhargava V., Lenfant B., Perret C., Pascual M.H., Sultan E., Montay G. (2002). Lack of effect of food on the bioavailability of a new ketolide antibacterial, telithromycin. Scand. J. Infect. Dis..

[151] Ortiz R.A., Calafatti S.A., Moraes L.A., Deguer M., Ecclissato C.C., Marchioretto M.A., Ribeiro M.L., Bernasconi G., Pedrazzoli J. (2007). Effect of Helicobacter pylori infection and acid blockade by lansoprazole on clarithromycin bioavailability. Braz. J. Med. Biol. Res..

[152] Chiu L.M., Menhinick A.M., Johnson P.W., Amsden G.W. (2002). Pharmacokinetics of intravenous azithromycin and ceftriaxone when administered alone and concurrently to healthy volunteers. J. Antimicrob. Chemother..

[153] Ngo L.Y., Yogev R., Dankner W.M., Hughes W.T., Burchett S., Xu J., Sadler B., Unadkat J.D. (1999). Pharmacokinetics of azithromycin administered alone and with atovaquone in human immunodeficiency virus-infected children. The ACTG 254 Team. Antimicrob. Agents Chemother..

[154] Cook J.A., Randinitis E.J., Bramson C.R., Wesche D.L. (2006). Lack of a pharmacokinetic interaction between azithromycin and chloroquine. Am. J. Trop. Med. Hyg..

[155] Amsden G., Flaherty J., Luke D. (2001). Lack of an effect of azithromycin on the disposition of zidovudine and dideoxyinosine in HIV-infected patients. J. Clin. Pharmacol..

[156] Apseloff G., Foulds G., LaBoy-Goral L., Willavize S., Vincent J. (1998). Comparison of azithromycin and clarithromycin in their interactions with rifabutin in healthy volunteers. J. Clin. Pharmacol..

[157] Harris S., Hilligoss D.M., Colangelo P.M., Eller M., Okerholm R. (1995). Azithromycin and terfenadine: lack of drug interaction. Clin. Pharmacol. Ther..

[158] Hagelberg N.M., Peltoniemi M.A., Saari T.I., Kurkinen K.J., Laine K., Neuvonen P.J., Olkkola K.T. (2010). Clarithromycin, a potent inhibitor of CYP3A, greatly increases exposure to oral S-ketamine. Eur. J. Pain.

[159] Jokinen M.J., Ahonen J., Neuvonen P.J., Olkkola K.T. (2001). Effect of clarithromycin and itraconazole on the pharmacokinetics of ropivacaine. Pharmacol. Toxicol..

[160] Olkkola K.T., Isohanni M.H., Hamunen K., Neuvonen P.J. (2005). The effect of erythromycin and fluvoxamine on the pharmacokinetics of intravenous lidocaine. Anesth. Analg..

[161] Isohanni M.H., Neuvonen P.J., Olkkola K.T. (1999). Effect of erythromycin and itraconazole on the pharmacokinetics of oral lignocaine. Pharmacol. Toxicol..

[162] Isohanni M.H., Neuvonen P.J., Palkama V.J., Olkkola K.T. (1998). Effect of erythromycin and itraconazole on the pharmacokinetics of intravenous lignocaine. Eur. J. Clin. Pharmacol..

[163] Brophy D.F., Israel D.S., Pastor A., Gillotin C., Chittick G.E., Symonds W.T., Lou Y., Sadler B.M., Polk R.E. (2000). Pharmacokinetic interaction between amprenavir and clarithromycin in healthy male volunteers. Antimicrob. Agents Chemother..

[164] Ouellet D., Hsu A., Granneman G.R., Carlson G., Cavanaugh J., Guenther H., Leonard J.M. (1998). Pharmacokinetic interaction between ritonavir and clarithromycin. Clin. Pharmacol. Ther..

[165] Boruchoff S.E., Sturgill M.G., Grasing K.W., Seibold J.R., McCrea J., Winchell G.A., Kusma S.E., Deutsch P.J. (2000). The steady-state disposition of indinavir is not altered by the concomitant administration of clarithromycin. Clin. Pharmacol. Ther..

[166] Sekar V.J., Spinosa-Guzman S., De Paepe E., De Pauw M., Vangeneugden T., Lefebvre E., Hoetelmans R.M. (2008). Darunavir/ritonavir pharmacokinetics following coadministration with clarithromycin in healthy volunteers. J. Clin. Pharmacol..

[167] Amsden G.W., Kuye O., Wei G.C. (2002). A study of the interaction potential of azithromycin and clarithromycin with atorvastatin in healthy volunteers. J. Clin. Pharmacol..

[168] Jacobson T.A. (2004). Comparative pharmacokinetic interaction profiles of pravastatin, simvastatin, and atorvastatin when coadministered with cytochrome P450 inhibitors. Am. J. Cardiol..

[169] Kantola T., Kivisto K.T., Neuvonen P.J. (1998). Erythromycin and verapamil considerably increase serum simvastatin and simvastatin acid concentrations. Clin. Pharmacol. Ther..

[170] Cooper K.J., Martin P.D., Dane A.L., Warwick M.J., Raza A., Schneck D.W. (2003). The effect of erythromycin on the pharmacokinetics of rosuvastatin. Eur. J. Clin. Pharmacol..

[171] Hassan-Alin M., Andersson T., Niazi M., Liljeblad M., Persson B.A., Rohss K. (2006). Studies on drug interactions between esomeprazole, amoxicillin and clarithromycin in healthy subjects. Int. J. Clin. Pharmacol. Ther..

[172] Miura M., Tada H., Yasui-Furukori N., Uno T., Sugawara K., Tateishi T., Suzuki T. (2005). Effect of clarithromycin on the enantioselective disposition of lansoprazole in relation to CYP2C19 genotypes. Chirality.

[173] Saito M., Yasui-Furukori N., Uno T., Takahata T., Sugawara K., Munakata A., Tateishi T. (2005). Effects of clarithromycin on lansoprazole pharmacokinetics between CYP2C19 genotypes. Br. J. Clin. Pharmacol..

[174] Mainz D., Borner K., Koeppe P., Kotwas J., Lode H. (2002). Pharmacokinetics of lansoprazole, amoxicillin and clarithromycin after simultaneous and single administration. J. Antimicrob. Chemother..

[175] Ushiama H., Echizen H., Nachi S., Ohnishi A. (2002). Dose-dependent inhibition of CYP3A activity by clarithromycin during Helicobacter pylori eradication therapy assessed by changes in plasma lansoprazole levels and partial cortisol clearance to 6beta-hydroxycortisol. Clin. Pharmacol. Ther..

[176] Calabresi L., Pazzucconi F., Ferrara S., Di Paolo A., Tacca M.D., Sirtori C. (2004). Pharmacokinetic interactions between omeprazole/pantoprazole and clarithromycin in health volunteers. Pharmacol. Res..

[177] Furuta T., Ohashi K., Kobayashi K., Iida I., Yoshida H., Shirai N., Takashima M., Kosuge K., Hanai H., Chiba K., Ishizaki T., Kaneko E. (1999). Effects of clarithromycin on the metabolism of omeprazole in relation to CYP2C19 genotype status in humans. Clin. Pharmacol. Ther..

[178] Gustavson L.E., Kaiser J.F., Edmonds A.L., Locke C.S., DeBartolo M.L., Schneck D.W. (1995). Effect of omeprazole on concentrations of clarithromycin in plasma and gastric tissue at steady state. Antimicrob. Agents Chemother..

[179] Pedrazzoli J., Calafatti S.A., Ortiz R.A., Dias F.E., Deguer M., Mendes F.D., Bento A.P., Pereira A.A., Piovesana H., Ferraz J.G., Lerner F., de Nucci G. (2001). Transfer of clarithromycin to gastric juice is enhanced by omeprazole in Helicobacter pylori-infected individuals. Scand. J. Gastroenterol..

[180] Shimizu M., Uno T., Yasui-Furukori N., Sugawara K., Tateishi T. (2006). Effects of clarithromycin and verapamil on rabeprazole pharmacokinetics between CYP2C19 genotypes. Eur. J. Clin. Pharmacol..

[181] Kees F., Holstege A., Ittner K.P., Zimmermann M., Lock G., Scholmerich J., Grobecker H. (2000). Pharmacokinetic interaction between proton pump inhibitors and roxithromycin in volunteers. Aliment. Pharmacol. Ther..

[182] Zapater P., Reus S., Tello A., Torrus D., Perez-Mateo M., Horga J.F. (2002). A prospective study of the clarithromycin-digoxin interaction in elderly patients. J. Antimicrob. Chemother..

[183] Tsutsumi K., Kotegawa T., Kuranari M., Otani Y., Morimoto T., Matsuki S., Nakano S. (2002). The effect of erythromycin and clarithromycin on the pharmacokinetics of intravenous digoxin in healthy volunteers. J. Clin. Pharmacol..

[184] Tanaka H., Matsumoto K., Ueno K., Kodama M., Yoneda K., Katayama Y., Miyatake K. (2003). Effect of clarithromycin on steady-state digoxin concentrations. Ann. Pharmacother..

[185] Rengelshausen J., Goggelmann C., Burhenne J., Riedel K.D., Ludwig J., Weiss J., Mikus G., Walter-Sack I., Haefeli W.E. (2003). Contribution of increased oral bioavailability and reduced nonglomerular renal clearance of digoxin to the digoxin-clarithromycin interaction. Br. J. Clin. Pharmacol..

[186] Demolis J.L., Strabach S., Vacheron F., Funck-Brentano C. (2005). Assessment of the effect of a single oral dose of telithromycin on sotalol-induced qt interval prolongation in healthy women. Br. J. Clin. Pharmacol..

[187] Schwarz U.I., Gramatte T., Krappweis J., Oertel R., Kirch W. (2000). P-glycoprotein inhibitor erythromycin increases oral bioavailability of talinolol in humans. Int. J. Clin. Pharmacol. Ther..

[188] Bailey D.G., Bend J.R., Arnold J.M., Tran L.T., Spence J.D. (1996). Erythromycin-felodipine interaction: magnitude, mechanism, and comparison with grapefruit juice. Clin. Pharmacol. Ther..

[189] Lilja J.J., Niemi M., Fredrikson H., Neuvonen P.J. (2007). Effects of clarithromycin and grapefruit juice on the pharmacokinetics of glibenclamide. Br. J. Clin. Pharmacol..

[190] Jayasagar G., Dixit A.A., Kishan V., Rao Y.M. (2000). Effect of clarithromycin on the pharmacokinetics of tolbutamide. Drug Metabol. Drug Interact..

[191] Fleishaker J.C., Phillips J.P. (1991). Evaluation of a potential interaction between erythromycin and glyburide in diabetic volunteers. J. Clin. Pharmacol..

[192] Carr R.A., Edmonds A., Shi H., Locke C.S., Gustavson L.E., Craft J.C., Harris S.I., Palmer R. (1998). Steady-state pharmacokinetics and electrocardiographic pharmacodynamics of clarithromycin and loratadine after individual or concomitant administration. Antimicrob. Agents Chemother..

[193] Hanrahan J.P., Choo P.W., Carlson W., Greineder D., Faich G.A., Platt R. (1995). Terfenadine-associated ventricular arrhythmias and QTc interval prolongation. A retrospective cohort comparison with other antihistamines among members of a health maintenance organization. Ann. Epidemiol..

[194] Petri N., Borga O., Nyberg L., Hedeland M., Bondesson U., Lennernas H. (2006). Effect of erythromycin on the absorption of fexofenadine in the jejunum, ileum and colon determined using local intubation in healthy volunteers. Int. J. Clin. Pharmacol. Ther..

[195] Banfield C., Hunt T., Reyderman L., Statkevich P., Padhi D., Affrime M. (2002). Lack of clinically relevant interaction between desloratadine and erythromycin. Clin. Pharmacokinet..

[196] Brannan M.D., Reidenberg P., Radwanski E., Shneyer L., Lin C.C., Cayen M.N., Affrime M.B. (1995). Loratadine administered concomitantly with erythromycin: pharmacokinetic and electrocardiographic evaluations. Clin. Pharmacol. Ther..

[197] Yeates R.A., Laufen H., Zimmermann T. (1996). Interaction between midazolam and clarithromycin: comparison with azithromycin. Int. J. Clin. Pharmacol. Ther..

[198] Zimmermann T., Yeates R.A., Laufen H., Scharpf F., Leitold M., Wildfeuer A. (1996). Influence of the antibiotics erythromycin and azithromycin on the pharmacokinetics and pharmacodynamics of midazolam. Arzneimittelforschung.

[199] Backman J.T., Olkkola K.T., Neuvonen P.J. (1995). Azithromycin does not increase plasma concentrations of oral midazolam. Int. J. Clin. Pharmacol. Ther..

[200] Mattila M.J., Vanakoski J., Idanpaan-Heikkila J.J. (1994). Azithromycin does not alter the effects of oral midazolam on human performance. Eur. J. Clin. Pharmacol..

[201] Yasui N., Otani K., Kaneko S., Ohkubo T., Osanai T., Sugawara K., Chiba K., Ishizaki T. (1996). A kinetic and dynamic study of oral alprazolam with and without erythromycin in humans: in vivo evidence for the involvement of CYP3A4 in alprazolam metabolism. Clin. Pharmacol. Ther..

[202] Luurila H., Olkkola K.T., Neuvonen P.J. (1996). Interaction between erythromycin and the benzodiazepines diazepam and flunitrazepam. Pharmacol. Toxicol..

[203] Luurila H., Olkkola K.T., Neuvonen P.J. (1995). Interaction between erythromycin and nitrazepam in healthy volunteers. Pharmacol. Toxicol..

[204] Phillips J.P., Antal E.J., Smith R.B. (1986). A pharmacokinetic drug interaction between erythromycin and triazolam. J. Clin. Psychopharmacol..

[205] Aranko K., Luurila H., Backman J.T., Neuvonen P.J., Olkkola K.T. (1994). The effect of erythromycin on the pharmacokinetics and pharmacodynamics of zopiclone. Br. J. Clin. Pharmacol..

[206] Luurila H., Olkkola K.T., Neuvonen P.J. (1994). Lack of interaction of erythromycin with temazepam. Ther. Drug Monit..

[207] Okudaira T., Kotegawa T., Imai H., Tsutsumi K., Nakano S., Ohashi K. (2007). Effect of the treatment period with erythromycin on cytochrome P450 3A activity in humans. J. Clin. Pharmacol..

[208] Olkkola K.T., Aranko K., Luurila H., Hiller A., Saarnivaara L., Himberg J.J., Neuvonen P.J. (1993). A potentially hazardous interaction between erythromycin and midazolam. Clin. Pharmacol. Ther..

[209] Backman J.T., Aranko K., Himberg J.J., Olkkola K.T. (1994). A pharmacokinetic interaction between roxithromycin and midazolam. Eur. J. Clin. Pharmacol..

[210] Quinney S.K., Haehner B.D., Rhoades M.B., Lin Z., Gorski J.C., Hall S.D. (2008). Interaction between midazolam and clarithromycin in the elderly. Br. J. Clin. Pharmacol..

[211] Gupta S.K., Bakran A., Johnson R.W., Rowland M. (1989). Cyclosporin-erythromycin interaction in renal transplant patients. Br. J. Clin. Pharmacol..

[212] Kovarik J.M., Beyer D., Bizot M.N., Jiang Q., Shenouda M., Schmouder R.L. (2005). Effect of multiple-dose erythromycin on everolimus pharmacokinetics. Eur. J. Clin. Pharmacol..

[213] Bachmann K., Jauregui L., Chandra R., Thakker K. (2003). Influence of a 3-day regimen of azithromycin on the disposition kinetics of cyclosporine A in stable renal transplant patients. Pharmacol. Res..

[214] Vernillet L., Bertault-Peres P., Berland Y., Barradas J., Durand A., Olmer M. (1989). Lack of effect of spiramycin on cyclosporin pharmacokinetics. Br. J. Clin. Pharmacol..

[215] Paulsen O., Nilsson L.G., Saint-Salvi B., Manuel C., Lunell E. (1988). No effect of roxithromycin on pharmacokinetic or pharmacodynamic properties of warfarin and its enantiomers. Pharmacol. Toxicol..

[216] Gillum J.G., Israel D.S., Scott R.B., Climo M.W., Polk R.E. (1996). Effect of combination therapy with ciprofloxacin and clarithromycin on theophylline pharmacokinetics in healthy volunteers. Antimicrob. Agents Chemother..

[217] Bartolucci L., Gradoli C., Vincenzi V., Iapadre M., Valori C. (1984). Macrolide antibiotics and serum theophylline levels in relation to the severity of respiratory impairment: a comparison between the effects of erythromycin and josamycin. Chemioterapia.

[218] May D.C., Jarboe C.H., Ellenburg D.T., Roe E.J., Karibo J. (1982). The effects of erythromycin on theophylline elimination in normal males. J. Clin. Pharmacol..

[219] Prince R.A., Wing D.S., Weinberger M.M., Hendeles L.S., Riegelman S. (1981). Effect of erythromycin on theophylline kinetics. J. Allergy Clin. Immunol..

[220] Renton K.W., Gray J.D., Hung O.R. (1981). Depression of theophylline elimination by erythromycin. Clin. Pharmacol. Ther..

[221] Hashiguchi K., Niki Y., Soejima R. (1992). Roxithromycin does not raise serum theophylline levels. Chest.

[222] Fost D.A., Leung D.Y., Martin R.J., Brown E.E., Szefler S.J., Spahn J.D. (1999). Inhibition of methylprednisolone elimination in the presence of clarithromycin therapy. J. Allergy Clin. Immunol..

[223] Kivisto K.T., Lamberg T.S., Kantola T., Neuvonen P.J. (1997). Plasma buspirone concentrations are greatly increased by erythromycin and itraconazole. Clin. Pharmacol. Ther..

[224] Hagg S., Spigset O., Mjorndal T., Granberg K., Persbo-Lundqvist G., Dahlqvist R. (1999). Absence of interaction between erythromycin and a single dose of clozapine. Eur. J. Clin. Pharmacol..

[225] Orlando R., De Martin S., Pegoraro P., Quintieri L., Palatini P. (2009). Irreversible CYP3A inhibition accompanied by plasma protein-binding displacement: a comparative analysis in subjects with normal and impaired liver function. Clin. Pharmacol. Ther..

[226] Nosten F., ter Kuile F., Thwai K.L., Maelankirri L., White N.J. (1993). Spiramycin does not potentiate quinine treatment of falciparum malaria in pregnancy. Trans. R. Soc. Trop. Med. Hyg..

[227] Keranen T., Jolkkonen J., Jensen P.K., Menge G.P., Andersson P. (1992). Absence of interaction between oxcarbazepine and erythromycin. Acta Neurol. Scand..

[228] Milne R.W., Coulthard K., Nation R.L., Penna A.C., Roberts G., Sansom L.N. (1988). Lack of effect of erythromycin on the pharmacokinetics of single oral doses of phenytoin. Br. J. Clin. Pharmacol..

[229] Thomsen M.S., Groes L., Agerso H., Kruse T. (1998). Lack of pharmacokinetic interaction between tiagabine and erythromycin. J. Clin. Pharmacol..

[230] Gronlund J., Saari T., Hagelberg N., Martikainen I.K., Neuvonen P.J., Olkkola K.T., Laine K. (2010). Effect of telithromycin on the pharmacokinetics and pharmacodynamics of oral oxycodone. J. Clin. Pharmacol..

[231] Amsden G.W., Gregory T.B., Michalak C.A., Glue P., Knirsch C.A. (2007). Pharmacokinetics of azithromycin and the combination of ivermectin and albendazole when administered alone and concurrently in healthy volunteers. Am. J. Trop. Med. Hyg..

[232] Nakatsuka A., Nagai M., Yabe H., Nishikawa N., Nomura T., Moritoyo H., Moritoyo T., Nomoto M. (2006). Effect of clarithromycin on the pharmacokinetics of cabergoline in healthy controls and in patients with Parkinson's disease. J. Pharmacol. Sci..

[233] Lahu G., Huennemeyer A., Herzog R., McCracken N., Hermann R., Elmlinger M., Zech K. (2009). Effect of repeated dose of erythromycin on the pharmacokinetics of roflumilast and roflumilast N-oxide. Int. J. Clin. Pharmacol. Ther..

[234] Amato C.S., Wang R.Y., Wright R.O., Linakis J.G. (2004). Evaluation of promotility agents to limit the gut bioavailability of extended-release acetaminophen. J. Toxicol. Clin. Toxicol..

[235] Callreus T., Lundahl J., Hoglund P., Bengtsson P. (1999). Changes in gastrointestinal motility influence the absorption of desmopressin. Eur. J. Clin. Pharmacol..

[236] Pesco-Koplowitz L., Hassell A., Lee P., Zhou H., Hall N., Wiesinger B., Mechlinski W., Grover M., Hunt T., Smith R., Travers S. (1999). Lack of effect of erythromycin and ketoconazole on the pharmacokinetics and pharmacodynamics of steady-state intranasal levocabastine. J. Clin. Pharmacol..

[237] Shah A., Liu M.C., Vaughan D., Heller A.H. (1999). Oral bioequivalence of three ciprofloxacin formulations following single-dose administration: 500 mg tablet compared with 500 mg/10 mL or 500 mg/5 mL suspension and the effect of food on the absorption of ciprofloxacin oral suspension. J. Antimicrob. Chemother..

[238] Kawakami J., Matsuse T., Kotaki H., Seino T., Fukuchi Y., Orimo H., Sawada Y., Iga T. (1994). The effect of food on the interaction of ofloxacin with sucralfate in healthy volunteers. Eur. J. Clin. Pharmacol..

[239] Healy D.P., Brodbeck M.C., Clendening C.E. (1996). Ciprofloxacin absorption is impaired in patients given enteral feedings orally and via gastrostomy and jejunostomy tubes. Antimicrob. Agents Chemother..

[240] Mueller B.A., Brierton D.G., Abel S.R., Bowman L. (1994). Effect of enteral feeding with ensure on oral bioavailabilities of ofloxacin and ciprofloxacin. Antimicrob. Agents Chemother..

[241] Neuhofel A.L., Wilton J.H., Victory J.M., Hejmanowsk L.G., Amsden G.W. (2002). Lack of bioequivalence of ciprofloxacin when administered with calcium-fortified orange juice: a new twist on an old interaction. J. Clin. Pharmacol..

[242] Neuvonen P.J., Kivisto K.T., Lehto P. (1991). Interference of dairy products with the absorption of ciprofloxacin. Clin. Pharmacol. Ther..

[243] Kanji S., McKinnon P.S., Barletta J.F., Kruse J.A., Devlin J.W. (2003). Bioavailability of gatifloxacin by gastric tube administration with and without concomitant enteral feeding in critically ill patients. Crit Care Med..

[244] Wallace A.W., Victory J.M., Amsden G.W. (2003). Lack of bioequivalence of gatifloxacin when coadministered with calcium-fortified orange juice in healthy volunteers. J. Clin. Pharmacol..

[245] Nakashima M., Uematsu T., Kosuge K., Kusajima H., Ooie T., Masuda Y., Ishida R., Uchida H. (1995). Single- and multiple-dose pharmacokinetics of AM-1155, a new 6-fluoro-8-methoxy quinolone, in humans. Antimicrob. Agents Chemother..

[246] Lee L.J., Hafkin B., Lee I.D., Hoh J., Dix R. (1997). Effects of food and sucralfate on a single oral dose of 500 milligrams of levofloxacin in healthy subjects. Antimicrob. Agents Chemother..

[247] Amsden G.W., Whitaker A.M., Johnson P.W. (2003). Lack of bioequivalence of levofloxacin when coadministered with a mineral-fortified breakfast of juice and cereal. J. Clin. Pharmacol..

[248] Wallace A.W., Victory J.M., Amsden G.W. (2003). Lack of bioequivalence when levofloxacin and calcium-fortified orange juice are coadministered to healthy volunteers. J. Clin. Pharmacol..

[249] Burkhardt O., Stass H., Thuss U., Borner K., Welte T. (2005). Effects of enteral feeding on the oral bioavailability of moxifloxacin in healthy volunteers. Clin. Pharmacokinet..

[250] Stass H., Kubitza D. (2001). Effects of dairy products on the oral bioavailability of moxifloxacin, a novel 8-methoxyfluoroquinolone, in healthy volunteers. Clin. Pharmacokinet..

[251] Kara M., Hasinoff B.B., McKay D.W., Campbell N.R. (1991). Clinical and chemical interactions between iron preparations and ciprofloxacin. Br. J. Clin. Pharmacol..

[252] Lehto P., Kivisto K.T., Neuvonen P.J. (1994). The effect of ferrous sulphate on the absorption of norfloxacin, ciprofloxacin and ofloxacin. Br. J. Clin. Pharmacol..

[253] Stass H., Schuhly U., Moller J.G., Delesen H. (2001). Effects of sucralfate on the oral bioavailability of moxifloxacin, a novel 8-methoxyfluoroquinolone, in healthy volunteers. Clin. Pharmacokinet..

[254] Lehto P., Kivisto K.T. (1994). Different effects of products containing metal ions on the absorption of lomefloxacin. Clin. Pharmacol. Ther..

[255] Kays M.B., Overholser B.R., Mueller B.A., Moe S.M., Sowinski K.M. (2003). Effects of sevelamer hydrochloride and calcium acetate on the oral bioavailability of ciprofloxacin. Am. J. Kidney Dis..

[256] Pletz M.W., Petzold P., Allen A., Burkhardt O., Lode H. (2003). Effect of calcium carbonate on bioavailability of orally administered gemifloxacin. Antimicrob. Agents Chemother..

[257] Pai M.P., Allen S.E., Amsden G.W. (2006). Altered steady state pharmacokinetics of levofloxacin in adult cystic fibrosis patients receiving calcium carbonate. J. Cyst. Fibros..

[258] Stass H., Wandel C., Delesen H., Moller J.G. (2001). Effect of calcium supplements on the oral bioavailability of moxifloxacin in healthy male volunteers. Clin. Pharmacokinet..

[259] Sanchez Navarro A., Martinez Cabarga M., Dominguez-Gil Hurle A. (1994). Oral absorption of ofloxacin administered together with aluminum. Antimicrob. Agents Chemother..

[260] Quain R.D., Barton T.D., Fishman N.O., Weiner M.G., Lautenbach E. (2005). Coadministration of oral levofloxacin with agents that impair its absorption: potential impact on emergence of resistance. Int. J. Antimicrob. Agents.

[261] How P.P., Fischer J.H., Arruda J.A., Lau A.H. (2007). Effects of lanthanum carbonate on the absorption and oral bioavailability of ciprofloxacin. Clin. J. Am. Soc. Nephrol..

[262] Nix D.E., Watson W.A., Lener M.E., Frost R.W., Krol G., Goldstein H., Lettieri J., Schentag J.J. (1989). Effects of aluminum and magnesium antacids and ranitidine on the absorption of ciprofloxacin. Clin. Pharmacol. Ther..

[263] Maesen F.P., Davies B.I., Geraedts W.H., Sumajow C.A. (1987). Ofloxacin and antacids. J. Antimicrob. Chemother..

[264] Lober S., Ziege S., Rau M., Schreiber G., Mignot A., Koeppe P., Lode H. (1999). Pharmacokinetics of gatifloxacin and interaction with an antacid containing aluminum and magnesium. Antimicrob. Agents Chemother..

[265] Stass H., Bottcher M.F., Ochmann K. (2001). Evaluation of the influence of antacids and H2 antagonists on the absorption of moxifloxacin after oral administration of a 400 mg dose to healthy volunteers. Clin. Pharmacokinet..

[266] Stuht H., Lode H., Koeppe P., Rost K.L., Schaberg T. (1995). Interaction study of lomefloxacin and ciprofloxacin with omeprazole and comparative pharmacokinetics. Antimicrob. Agents Chemother..

[267] Washington C., Hou E., Hughes N., Berner B. (2006). Effect of omeprazole on bioavailability of an oral extended-release formulation of ciprofloxacin. Am. J. Health Syst. Pharm..

[268] Garrelts J.C., Godley P.J., Peterie J.D., Gerlach E.H., Yakshe C.C. (1990). Sucralfate significantly reduces ciprofloxacin concentrations in serum. Antimicrob. Agents Chemother..

[269] Van Slooten A.D., Nix D.E., Wilton J.H., Love J.H., Spivey J.M., Goldstein H.R. (1991). Combined use of ciprofloxacin and sucralfate. DICP.

[270] Nix D.E., Watson W.A., Handy L., Frost R.W., Rescott D.L., Goldstein H.R. (1989). The effect of sucralfate pretreatment on the pharmacokinetics of ciprofloxacin. Pharmacotherapy.

[271] Lehto P., Kivisto K.T. (1994). Effect of sucralfate on absorption of norfloxacin and ofloxacin. Antimicrob. Agents Chemother..

[272] Rambout L., Sahai J., Gallicano K., Oliveras L., Garber G. (1994). Effect of bismuth subsalicylate on ciprofloxacin bioavailability. Antimicrob. Agents Chemother..

[273] Knupp C.A., Barbhaiya R.H. (1997). A multiple-dose pharmacokinetic interaction study between didanosine (Videx) and ciprofloxacin (Cipro) in male subjects seropositive for HIV but asymptomatic. Biopharm. Drug Dispos..

[274] Sahai J., Gallicano K., Oliveras L., Khaliq S., Hawley-Foss N., Garber G. (1993). Cations in the didanosine tablet reduce ciprofloxacin bioavailability. Clin. Pharmacol. Ther..

[275] Damle B.D., Kaul S., Behr D., Knupp C. (2002). Bioequivalence of two formulations of didanosine, encapsulated enteric-coated beads and buffered tablet, in healthy volunteers and HIV-infected subjects. J. Clin. Pharmacol..

[276] Ilo C.E., Ezejiofor N.A., Agbakoba N., Brown S.A., Maduagwuna C.A., Agbasi P.U., Orisakwe O.E. (2008). Effect of chloroquine on the urinary excretion of ciprofloxacin. Am. J. Ther..

[277] Ilo C.E., Ilondu N.A., Okwoli N., Brown S.A., Elo-Ilo J.C., Agbasi P.U., Orisakwe O.E. (2006). Effect of chloroquine on the bioavailability of ciprofloxacin in humans. Am. J. Ther..

[278] Marcelin-Jimenez G., Angeles A.P., Martinez-Rossier L., Fernandez S.A. (2006). Ciprofloxacin bioavailability is enhanced by oral co-administration with phenazopyridine: a pharmacokinetic study in a Mexican population. Clin. Drug Investig..

[279] Chien S.C., Rogge M.C., Williams R.R., Natarajan J., Wong F., Chow A.T. (2002). Absence of a pharmacokinetic interaction between digoxin and levofloxacin. J. Clin. Pharm. Ther..

[280] Lindenbaum J., Rund D.G., Butler V.P., Tse-Eng D., Saha J.R. (1981). Inactivation of digoxin by the gut flora: reversal by antibiotic therapy. N. Engl. J. Med..

[281] Johnson R.D., Dorr M.B., Hunt T.L., Conway S., Talbot G.H. (1999). Pharmacokinetic interaction of sparfloxacin and digoxin. Clin. Ther..

[282] Vousden M., Allen A., Lewis A., Ehren N. (1999). Lack of pharmacokinetic interaction between gemifloxacin and digoxin in healthy elderly volunteers. Chemotherapy.

[283] Jaehde U., Sorgel F., Reiter A., Sigl G., Naber K.G., Schunack W. (1995). Effect of probenecid on the distribution and elimination of ciprofloxacin in humans. Clin. Pharmacol. Ther..

[284] Landersdorfer C.B., Kirkpatrick C.M., Kinzig M., Bulitta J.B., Holzgrabe U., Jaehde U., Reiter A., Naber K.G., Rodamer M., Sorgel F. (2010). Competitive inhibition of renal tubular secretion of ciprofloxacin and metabolite by probenecid. Br. J. Clin. Pharmacol..

[285] Landersdorfer C.B., Kirkpatrick C.M., Kinzig M., Bulitta J.B., Holzgrabe U., Drusano G.L., Sorgel F. (2009). Competitive inhibition of renal tubular secretion of gemifloxacin by probenecid. Antimicrob. Agents Chemother..

[286] Parker A.C., Preston T., Heaf D., Kitteringham N.R., Choonara I. (1994). Inhibition of caffeine metabolism by ciprofloxacin in children with cystic fibrosis as measured by the caffeine breath test. Br. J. Clin. Pharmacol..

[287] Harder S., Staib A.H., Beer C., Papenburg A., Stille W., Shah P.M. (1988). 4-quinolones inhibit biotransformation of caffeine. Eur. J. Clin. Pharmacol..

[288] Mahr G., Sorgel F., Granneman G.R., Kinzig M., Muth P., Patterson K., Fuhr U., Nickel P., Stephan U. (1992). Effects of temafloxacin and ciprofloxacin on the pharmacokinetics of caffeine. Clin. Pharmacokinet..

[289] Randinitis E.J., Alvey C.W., Koup J.R., Rausch G., Abel R., Bron N.J., Hounslow N.J., Vassos A.B., Sedman A.J. (2001). Drug interactions with clinafloxacin. Antimicrob. Agents Chemother..

[290] Healy D.P., Schoenle J.R., Stotka J., Polk R.E. (1991). Lack of interaction between lomefloxacin and caffeine in normal volunteers. Antimicrob. Agents Chemother..

[291] Raaska K., Neuvonen P.J. (2000). Ciprofloxacin increases serum clozapine and N-desmethylclozapine: a study in patients with schizophrenia. Eur. J. Clin. Pharmacol..

[292] Letsas K.P., Sideris A., Kounas S.P., Efremidis M., Korantzopoulos P., Kardaras F. (2006). Drug-induced QT interval prolongation after ciprofloxacin administration in a patient receiving olanzapine. Int. J. Cardiol..

[293] Markowitz J.S., DeVane C.L. (1999). Suspected ciprofloxacin inhibition of olanzapine resulting in increased plasma concentration. J. Clin. Psychopharmacol..

[294] Batty K.T., Davis T.M., Ilett K.F., Dusci L.J., Langton S.R. (1995). The effect of ciprofloxacin on theophylline pharmacokinetics in healthy subjects. Br. J. Clin. Pharmacol..

[295] Davis R.L., Quenzer R.W., Kelly H.W., Powell J.R. (1992). Effect of the addition of ciprofloxacin on theophylline pharmacokinetics in subjects inhibited by cimetidine. Ann. Pharmacother..

[296] Loi C.M., Parker B.M., Cusack B.J., Vestal R. (1993). Individual and combined effects of cimetidine and ciprofloxacin on theophylline metabolism in male nonsmokers. Br. J. Clin. Pharmacol..

[297] Loi C.M., Parker B.M., Cusack B.J., Vestal R.E. (1997). Aging and drug interactions. III. Individual and combined effects of cimetidine and cimetidine and ciprofloxacin on theophylline metabolism in healthy male and female nonsmokers. J. Pharmacol. Exp. Ther..

[298] Robson R.A., Begg E.J., Atkinson H.C., Saunders D.A., Frampton C.M. (1990). Comparative effects of ciprofloxacin and lomefloxacin on the oxidative metabolism of theophylline. Br. J. Clin. Pharmacol..

[299] Sano M., Kawakatsu K., Ohkita C., Yamamoto I., Takeyama M., Yamashina H., Goto M. (1988). Effects of enoxacin, ofloxacin and norfloxacin on theophylline disposition in humans. Eur. J. Clin. Pharmacol..

[300] Niki Y., Hashiguchi K., Miyashita N., Nakajima M., Matsushima T. (1999). Influence of gatifloxacin, a new quinolone antibacterial, on pharmacokinetics of theophylline. J. Infect. Chemother..

[301] Stass H., Kubitza D. (2001). Lack of pharmacokinetic interaction between moxifloxacin, a novel 8-methoxyfluoroquinolone, and theophylline. Clin. Pharmacokinet..

[302] Efthymiopoulos C., Bramer S.L., Maroli A., Blum B. (1997). Theophylline and warfarin interaction studies with grepafloxacin. Clin. Pharmacokinet..

[303] Davy M., Allen A., Bird N., Rost K.L., Fuder H. (1999). Lack of effect of gemifloxacin on the steady-state pharmacokinetics of theophylline in healthy volunteers. Chemotherapy.

[304] Dickens G.R., Wermeling D., Vincent J. (1997). Phase I pilot study of the effects of trovafloxacin (CP-99,219) on the pharmacokinetics of theophylline in healthy men. J. Clin. Pharmacol..

[305] Takagi K., Yamaki K., Nadai M., Kuzuya T., Hasegawa T. (1991). Effect of a new quinolone, sparfloxacin, on the pharmacokinetics of theophylline in asthmatic patients. Antimicrob. Agents Chemother..

[306] Isohanni M.H., Ahonen J., Neuvonen P.J., Olkkola K.T. (2005). Effect of ciprofloxin on the pharmacokinetics of intravenous lidocaine. Eur. J. Anaesthesiol..

[307] Jokinen M.J., Olkkola K.T., Ahonen J., Neuvonen P.J. (2003). Effect of ciprofloxacin on the pharmacokinetics of ropivacaine. Eur. J. Clin. Pharmacol..

[308] Bauer L.A., Black D.J., Lill J.S., Garrison J., Raisys V.A., Hooton T.M. (2005). Levofloxacin and ciprofloxacin decrease procainamide and N-acetylprocainamide renal clearances. Antimicrob. Agents Chemother..

[309] Granfors M.T., Backman J.T., Neuvonen M., Neuvonen P.J. (2004). Ciprofloxacin greatly increases concentrations and hypotensive effect of tizanidine by inhibiting its cytochrome P450 1A2-mediated presystemic metabolism. Clin. Pharmacol. Ther..

[310] Tan K.K., Trull A.K., Shawket S. (1989). Co-administration of ciprofloxacin and cyclosporin: lack of evidence for a pharmacokinetic interaction. Br. J. Clin. Pharmacol..

[311] Capone D., Tarantino G., Polichetti G., Kadilli I., Sabbatini M., Basile V., Carrano R., Nappi R., Federico S. (2010). Absence of pharmacokinetic interference of moxifloxacin on cyclosporine and tacrolimus in kidney transplant recipients. J. Clin. Pharmacol..

[312] Doose D.R., Walker S.A., Chien S.C., Williams R.R., Nayak R.K. (1998). Levofloxacin does not alter cyclosporine disposition. J. Clin. Pharmacol..

[313] Garber S.M., Pound M.W., Miller S.M. (2009). Hypoglycemia associated with the use of levofloxacin. Am. J. Health Syst. Pharm..

[314] Lin G., Hays D.P., Spillane L. (2004). Refractory hypoglycemia from ciprofloxacin and glyburide interaction. J. Toxicol. Clin. Toxicol..

[315] Roberge R.J., Kaplan R., Frank R., Fore C. (2000). Glyburide-ciprofloxacin interaction with resistant hypoglycemia. Ann. Emerg. Med..

[316] Herrlin K., Segerdahl M., Gustafsson L.L., Kalso E. (2000). Methadone, ciprofloxacin, and adverse drug reactions. Lancet.

[317] Lee J., Franz L., Goforth H.W. (2009). Serotonin syndrome in a chronic-pain patient receiving concurrent methadone, ciprofloxacin, and venlafaxine. Psychosomatics.

[318] Nair M.K., Patel K., Starer P.J. (2008). Ciprofloxacin-induced torsades de pointes in a methadone-dependent patient. Addiction.

[319] Orisakwe O.E., Agbasi P.U., Afonne O.J., Ofoefule S.I., Obi E., Orish C.N. (2001). Rifampicin pharmacokinetics with and without ciprofloxacin. Am. J. Ther..

[320] Dorani H., Schutzer K.M., Sarich T.C., Wall U., Logren U., Ohlsson L., Eriksson U.G. (2007). Pharmacokinetics and pharmacodynamics of the oral direct thrombin inhibitor ximelagatran co-administered with different classes of antibiotics in healthy volunteers. Eur. J. Clin. Pharmacol..

[321] Welling P.G., Dean S., Selen A., Kendall M.J., Wise R. (1979). Probenecid: an unexplained effect on cephalosporin pharmacology. Br. J. Clin. Pharmacol..

[322] Garton A.M., Rennie R.P., Gilpin J., Marrelli M., Shafran S.D. (1997). Comparison of dose doubling with probenecid for sustaining serum cefuroxime levels. J. Antimicrob. Chemother..

[323] Spina S.P., Dillon E.C. (2003). Effect of chronic probenecid therapy on cefazolin serum concentrations. Ann. Pharmacother..

[324] Davis R.L., Berman W., Wernly J.A., Kelly H.W. (1991). Warfarin-nafcillin interaction. J. Pediatr..

[325] Garg A., Mohammed M. (2009). Decreased INR response secondary to warfarin-flucloxacillin interaction. Ann. Pharmacother..

[326] Heilker G.M., Fowler J.W., Self T.H. (1994). Possible nafcillin-warfarin interaction. Arch. Intern. Med..

[327] Merwick A., Hannon N., Kelly P.J., O'Rourke K. (2010). Warfarin-flucloxacillin interaction presenting as cardioembolic ischemic stroke. Eur. J. Clin. Pharmacol..

[328] Taylor A.T., Pritchard D.C., Goldstein A.O., Fletcher J.L. (1994). Continuation of warfarin-nafcillin interaction during dicloxacillin therapy. J. Fam. Pract..

[329] Davydov L., Yermolnik M., Cuni L.J. (2003). Warfarin and amoxicillin/clavulanate drug interaction. Ann. Pharmacother..

[330] Soto J., Sacristan J.A., Alsar M.J., Fernandez-Viadero C., Verduga R. (1993). Probable acenocoumarol-amoxycillin interaction. Acta Haematol..

[331] Zhang Q., Simoneau G., Verstuyft C., Drouet L., Bal dit Sollier C., Alvarez J.C., Rizzo-Padoin N., Bergmann J.F., Becquemont L., Mouly S. (2011). Amoxicillin/clavulanic acid-warfarin drug interaction: a randomized controlled trial. Br. J. Clin. Pharmacol..

[332] Lang C.C., Jamal S.K., Mohamed Z., Mustafa M.R., Mustafa A.M., Lee T.C. (2003). Evidence of an interaction between nifedipine and nafcillin in humans. Br. J. Clin. Pharmacol..

[333] Kishore K., Raina A., Misra V., Jonas E. (1993). Acute verapamil toxicity in a patient with chronic toxicity: possible interaction with ceftriaxone and clindamycin. Ann. Pharmacother..

[334] Ronchera C.L., Hernandez T., Peris J.E., Torres F., Granero L., Jimenez N.V., Pla J.M. (1993). Pharmacokinetic interaction between high-dose methotrexate and amoxycillin. Ther. Drug Monit..

[335] Titier K., Lagrange F., Pehourcq F., Moore N., Molimard M. (2002). Pharmacokinetic interaction between high-dose methotrexate and oxacillin. Ther. Drug Monit..

[336] Zarychanski R., Wlodarczyk K., Ariano R., Bow E. (2006). Pharmacokinetic interaction between methotrexate and piperacillin/tazobactam resulting in prolonged toxic concentrations of methotrexate. J. Antimicrob. Chemother..

[337] Landersdorfer C.B., Kirkpatrick C.M., Kinzig M., Bulitta J.B., Holzgrabe U., Sorgel F. (2008). Inhibition of flucloxacillin tubular renal secretion by piperacillin. Br. J. Clin. Pharmacol..

[338] Thompson M.I., Russo M.E., Saxon B.J., Atkin-Thor E., Matsen J.M. (1982). Gentamicin inactivation by piperacillin or carbenicillin in patients with end-stage renal disease. Antimicrob. Agents Chemother..

[339] Halstenson C.E., Hirata C.A., Heim-Duthoy K.L., Abraham P.A., Matzke G.R. (1990). Effect of concomitant administration of piperacillin on the dispositions of netilmicin and tobramycin in patients with end-stage renal disease. Antimicrob. Agents Chemother..

[340] Konishi H., Goto M., Nakamoto Y., Yamamoto I., Yamashina H. (1983). Tobramycin inactivation by carbenicillin, ticarcillin, and piperacillin. Antimicrob. Agents Chemother..

[341] Rodondi L.C., Flaherty J.F., Schoenfeld P., Barriere S.L., Gambertoglio J.G. (1989). Influence of coadministration on the pharmacokinetics of mezlocillin and cefotaxime in healthy volunteers and in patients with renal failure. Clin. Pharmacol. Ther..

[342] Spriet I., Meersseman W., De Troy E., Wilmer A., Casteels M., Willems L. (2007). Meropenem-valproic acid interaction in patients with cefepime-associated status epilepticus. Am. J. Health Syst. Pharm..

[343] Fudio S., Carcas A., Pinana E., Ortega R. (2006). Epileptic seizures caused by low valproic acid levels from an interaction with meropenem. J. Clin. Pharm. Ther..

[344] Clause D., Decleire P.Y., Vanbinst R., Soyer A., Hantson P. (2005). Pharmacokinetic interaction between valproic acid and meropenem. Intensive Care Med..

[345] Santucci M., Parmeggiani A., Riva R. (2005). Seizure worsening caused by decreased serum valproate during meropenem therapy. J. Child Neurol..

[346] Coves-Orts F.J., Borras-Blasco J., Navarro-Ruiz A., Murcia-Lopez A., Palacios-Ortega F. (2005). Acute seizures due to a probable interaction between valproic acid and meropenem. Ann. Pharmacother..

[347] Nacarkucuk E., Saglam H., Okan M. (2004). Meropenem decreases serum level of valproic acid. Pediatr. Neurol..

[348] De Turck B.J., Diltoer M.W., Cornelis P.J., Maes V., Spapen H.D., Camu F., Huyghens L.P. (1998). Lowering of plasma valproic acid concentrations during concomitant therapy with meropenem and amikacin. J. Antimicrob. Chemother..

[349] Dasgupta A., Sperelakis A., Mason A., Dean R. (1997). Phenytoin-oxacillin interactions in normal and uremic sera. Pharmacotherapy.

[350] Franco-Bronson K., Gajwani P. (1999). Hypotension associated with intravenous haloperidol and imipenem. J. Clin. Psychopharmacol..

[351] Connor H. (2003). Serotonin syndrome after single doses of co-amoxiclav during treatment with venlafaxine. J. R. Soc. Med..

[352] Semel J.D., Allen N. (1991). Seizures in patients simultaneously receiving theophylline and imipenem or ciprofloxacin or metronidazole. South. Med. J..

[353] Portier H., Chalopin J.M., Freysz M., Tanter Y. (1980). Interaction between cephalosporins and alcohol. Lancet.

[354] Jayasagar G., Krishna Kumar M., Chandrasekhar K., Madhusudan Rao C., Madhusudan Rao Y. (2002). Effect of cephalexin on the pharmacokinetics of metformin in healthy human volunteers. Drug Metabol. Drug Interact..

[355] Dickinson L., Khoo S., Back D. (2008). Differences in the pharmacokinetics of protease inhibitors between healthy volunteers and HIV-infected persons. Curr. Opin. HIV. AIDS.

[356] Gomez C.M., Cordingly J.J., Palazzo M.G. (1999). Altered pharmacokinetics of ceftazidime in critically ill patients. Antimicrob. Agents Chemother..

[357] Stockley I. (2011). General Considerations and an outline survey of some basic interaction mechanisms. Stockley's Drug Interactions.

